# A Post-GWAS Analysis of the Shared Genetic Architecture Between COVID-19 and Coronary Artery Disease

**DOI:** 10.3390/ijms27094132

**Published:** 2026-05-05

**Authors:** Muhammad Sarfraz Ali, Waseem Haider, Sana Aziz, Anwaruddin Mohammad, Ani Manichaikul, Weibin Shi

**Affiliations:** 1 Department of Radiology and Medical Imaging, University of Virginia, Charlottesville, VA 22903, USA; sfj2zp@virginia.edu; 2 Department of Biosciences, COMSATS University, Islamabad 45550, Pakistan; 3 Department of Zoology, Faisalabad Campus, University of Education, Lahore 38000, Pakistan; sana.aziz1994@gmail.com; 4 Bioinformatics Core, University of Virginia School of Medicine, Charlottesville, VA 22903, USA; xdh9bx@virginia.edu; 5 Department of Genome Sciences, University of Virginia, Charlottesville, VA 22903, USA; am3xa@virginia.edu

**Keywords:** COVID-19, coronary artery disease, pleiotropy, proxitropy, GWAS, Mendelian randomization, GSMR, post-GWAS, *DMTN*, *PIWIL2*

## Abstract

An individual’s host genetics influence its susceptibility to both COVID-19 and coronary artery disease (CAD). We analyzed large-scale GWAS datasets encompassing 7.7 million SNPs to identify shared genetic architecture between the two diseases. We identified 24 pleiotropic risk loci for both COVID-19 and CAD, with three loci (1p31.1, 8p21.3, and 18q11.2) showing strong evidence for a single shared causal variant. Loci in the 8p21.3 and 18q11.2 regions showed a bidirectional causal association: COVID-19 to CAD or vice versa, while the 1p31.1 locus only showed a CAD to COVID-19 unilateral casual association in a Mendelian randomization analysis (GSMR). A fine mapping analysis of the three loci identified three lead pleiotropic variants (rs7515509, rs8192330, and rs4800403). The variant rs7515509 was spatially associated with *AK5, PIGK, USP33*, and *ZZZ3*; rs8192330 with *DMTN, PIWIL2*, and several other genes; and rs4800403 with *GATA6* and *CTAGE1*. Transcriptomic profiling of peripheral blood mononuclear cells (PBMCs) from COVID-19 patients validated proxitropic variants (rs8192330 and rs4800403) with distinct expression signatures and prioritized *DMTN* and *PIWIL2* as the likely causal genes. Overexpression of *DMTN* has been linked to the heme metabolism hallmark, disrupted iron distribution in COVID-19 patients with comorbid CAD, and subsequent stress erythropoiesis, oxidative stress, immunological dysfunction, and altered wound healing, while a lower expression of *PIWIL2* has been observed in the cytoplasmic translation and regulation of mRNA metabolism. In conclusion, we identified shared genetic components for COVID-19 and CAD and prioritized *DMTN* and *PIWIL2* as the likely causal genes for the observed shared genetic risk. COVID-19 may act as an acute stressor that unmask or accelerates underlying CAD.

## 1. Introduction

COVID-19, a global pandemic infectious disease caused by the SARS-CoV-2 virus, has infected 5.6 million people and resulted in over five million deaths as of 31 January 2022 [1]. It may progress to long COVID-19, with symptoms persisting for weeks, months, or even years after the initial acute phase of illness. Long COVID-19 is an often-debilitating illness that occurs in at least 10% of the cases or approximately 65 million individuals worldwide as of 2023 [2]. Beyond the respiratory symptoms, more than 200 symptoms have been identified, spanning multiple organ systems [2], specifically cardiovascular, neurological, pulmonary, and psychological ones [3]. Conversely, pre-existing obesity, heart failure, and ischemic heart disease are significant risk factors for increased susceptibility, severity of coronavirus infection, and the development of long COVID-19 [4]. Epidemiological data have revealed that COVID-19 infection is associated with a markedly increased rate of major adverse cardiovascular and thrombotic events within 2 years of infection [5,6,7,8,9,10]. The primary types of complications include myocarditis, acute coronary syndrome (ACS), hypotension, heart failure, shock, and sepsis [11]. Likely reasons for the increased risk include endothelial dysfunction [12,13], cytokine storms [14,15,16], leukocytes–platelets crosstalk [17,18,19], dyslipidemia [20,21], hyperglycemia [22,23], and oxidative stress [24].

Genome-wide association studies (GWAS) and subsequent meta-analysis have identified over 50 loci associated with the susceptibility and the severity of COVID-19 [25,26], including loci on chromosome 3p21 and the ABO locus on chromosome 9q34, which are associated with atherosclerotic plaque rupture and myocardial infarction [27,28]. Thus, there is a probability that the increased risk for major cardiovascular events in COVID-19 patients may stem from a shared genetic architecture. Specifically, the genetic loci *LZTFL1, ABO, ILRUN*, and *CACFD1* may simultaneously influence both COVID-19 severity and coronary artery disease (CAD) risk [29]. Subsequent studies have verified the significance of the ABO locus in the genetic interaction between COVID-19 and CAD [9,30]. However, the precise mechanisms and causal pathways modulating this interplay remain poorly understood. Here, we investigated the commonalities in the genetic architecture of COVID-19 and CAD to identify novel pleiotropic loci. By integrating cross-trait meta-analysis, generalized summary data-based Mendelian randomization (GSMR), and gene-based association testing through mBAT-Combo, we prioritized candidate genes. These were further validated using bulk RNA-seq-based expression profiling in COVID-19 patients and gene set enrichment analysis to elucidate the shared molecular pathology underlying both conditions. A schematic overview of this study is presented in Figure S1.

## 2. Results

### 2.1. Global Genetic Correlation Between COVID-19 and CAD

We used the linkage disequilibrium (LD) score regression to assess bivariate genetic correlations between CAD and three COVID-19 clinical phenotypes: (1) critically ill cases, (2) moderate to severe hospitalized cases, and (3) general SARS-CoV-2 reported cases. As shown in Table 1, significant genetic correlations were found between critically ill COVID-19 patients and CAD for both European (EUR) population (*R*^G^ = 0.1028; *P*_R_^G^ < 2.03 × 10^−7^) and South Asian (SAS) (*R*^G^ = 0.0938; *P*_R_^G^ < 5.70 × 10^−5^) ancestry including Lahore Punjabi in Pakistan. Moderate to severe hospitalized COVID-19 patients showed the strongest correlations (EUR: *R*^G^ = 0.1516; *P*_R_^G^ < 2.67 × 10^−17^; SAS: *R*^G^ = 0.1576; *P*_R_^G^ < 1.07 × 10^−11^). For general SARS-CoV-2 reported cases, correlations were also significant (EUR: *R*^G^ = 0.1219; *P*_R_^G^ < 4.042 × 10^−11^; SAS: *R*^G^ = 0.1344; *P*_R_^G^ < 3.68 × 10^−8^). Among the three COVID-19 categories, the moderate to severe hospitalized phenotype showed the strongest genetic association with CAD in both ancestries. These findings indicate that individuals with COVID-19 are at the greatest risk of developing CAD.

### 2.2. Coincident Loci for COVID-19 and CAD

We analyzed 7685,407 GWAS-curated SNPs identical to both GWAS datasets to identify coincident genomic risk loci for COVID-19 and CAD using colocalization analysis. Each locus containing lead SNPs and secondary signals within a 500 kb window was assessed using the Approximate Bayes Factors (ABF) method [31]. We identified 24 coincident risk loci significantly associated with both diseases, defined by a combined posterior probability (*PPH*_3_ + *PPH*_4_) of ≥ 0.7 (Table S1). Of these, 21 loci were associated with distinct causal variants for each disease, whereas only 3 loci showed evidence for a single shared pleiotropic variant (Table S1). Among the 21 coincident regions, loci harboring *SLC6A20, LZTFL1* (3p21.31), *IRF1, IL5* (5q31.1), *ABO* (9q34.2), and *IFNAR2* (21q22.11) were predominantly associated with COVID-19, while loci containing *CFDP1* (16q23.1), *COL1A1* (17q21.33), *LDLR* (19p13.2), *RSPH6A*, and *APOE* (19q13.32) were primarily linked to CAD (Table S1).

Subsequent HyPrColoc analysis provided strong statistical evidence for pleiotropy at 1p31.1, 8p21.3, and 18q11.2 loci, with posterior probabilities of ≥ 0.75 (Table S2). These loci are located at chromosomes 1:77,695,983–78,192,445 (1p31.1; *PP*_(HyPrColoc)_ = 0.85, *PIGK*) with lead signal rs7515509 (*p* < 2.94 × 10^−12^), 8:21,773,384–22,270,797 (8p21.3; *PP*_(HyPrColoc)_ = 0.90, *DMTN* and *PIWIL2*) with lead signal rs8192330 (*p* < 2.83 × 10^−7^), and 18:19,748,905–20,248,798 (18q11.2; *PP*_(HyPrColoc)_ = 0.97, *GATA6* and *CTAGE1*) with lead signal rs4800403 (*p* < 1.27 × 10^−7^).

**Figure 1:**
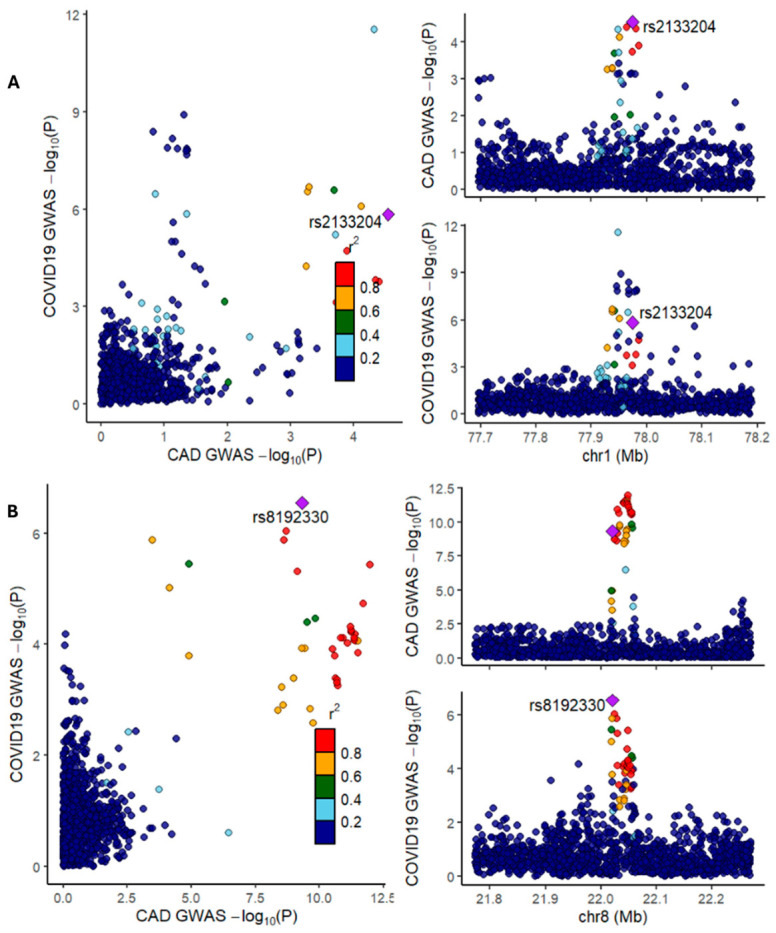

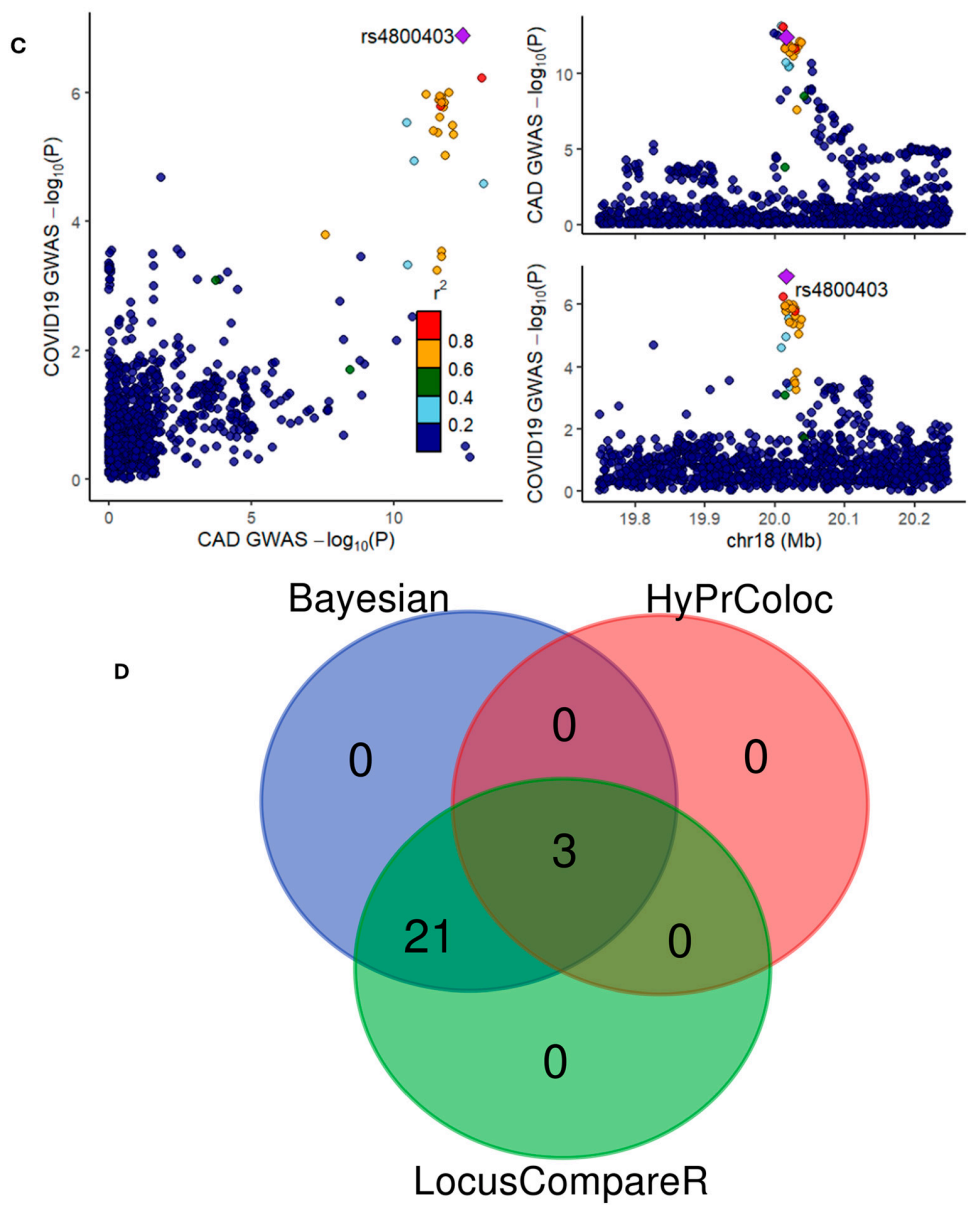
Visualization of GWAS-GWAS colocalization events and Venn Diagram. GWAS-GWAS LocusCompareR plots (**A**–**C**) where each dot represents a SNP in the region of interest (chr1:77,695,983–78,192,445 (1p31.1), chr8:21,773,384–22,270,797 (8p21.3), and chr18:19,748,905–20,248,798) (18q11.2) respectively) are shown. The labeled SNPs highlighted with a purple squares are the lead SNP (for both traits) and other SNPs are colored according to their LD (r^2^) with the lead SNPs present in their respective loci. The X and Y axes represent −log10 *p* values from CAD, and COVID-19 genome-wide association studies. (**D**) Venn diagram presents a systematic review of Bayesian, HyPrColoc, and LocusCompareR methods used for defining colocalization.

LocusCompare plots, which visualize the effect sizes of genetic variants from two GWAS datasets for a specific genomic region, confirmed colocalizations of lead variants for CAD and COVID-19 at these loci in both European and South Asian populations, including the Lahore Punjabi cohort in Pakistan (Figure 1A–C; Figure S2A–C). These results are summarized in a Venn diagram (Figure 1D; Table S3). The genetic variants at the three loci constituted a 99% credible set (Table S9).

### 2.3. Local Genetic Correlations Between COVID-19 and CAD

Given the significant genetic correlation between the moderate to severe hospitalized COVID-19 phenotype and CAD, we conducted local genetic correlation analyses at coincident pleiotropic loci. At the 1p31.1 locus, a significant correlation was observed in the EUR population (*R*^G^ = 0.6581, *P*_R_^G^ < 2 × 10^−4^), though no significant correlation was detected in the SAS population. At the 8p21.3 locus, strong inverse correlations were found for both EUR (*R*^G^ = −0.8112; *P*_R_^G^ < 5.45 × 10^−6^) and SAS ancestries (*R*^G^ = −0.8788; *P*_R_^G^ < 6.92 × 10^−8^). Finally, at the 18q11.2 locus, positive correlations between COVID-19 and CAD were maintained in both groups (*R*^G^ = 0.4767; *P*_R_^G^ < 7.87 × 10^−5^ for EUR population and *R*^G^ = 0.9226; *P*_R_^G^ < 7.92 × 10^−6^ for the SAS ancestry) (Table 1).

### 2.4. Coincident Pleotropic Signals Between COVID-19 and CAD

**Figure 2:**
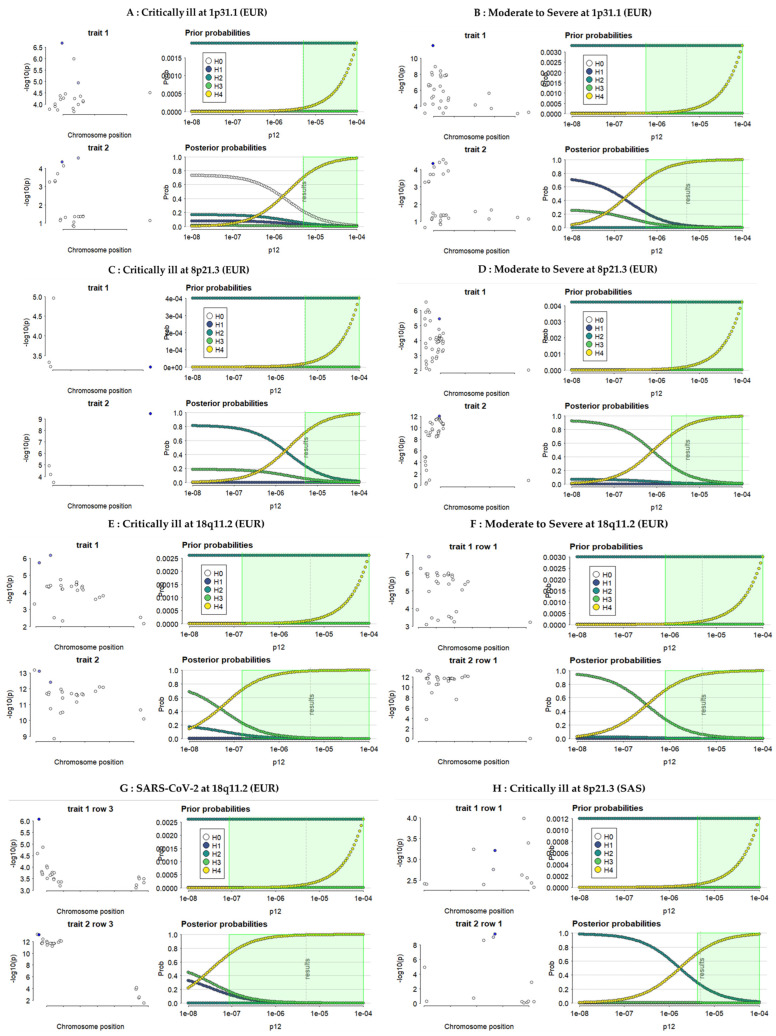

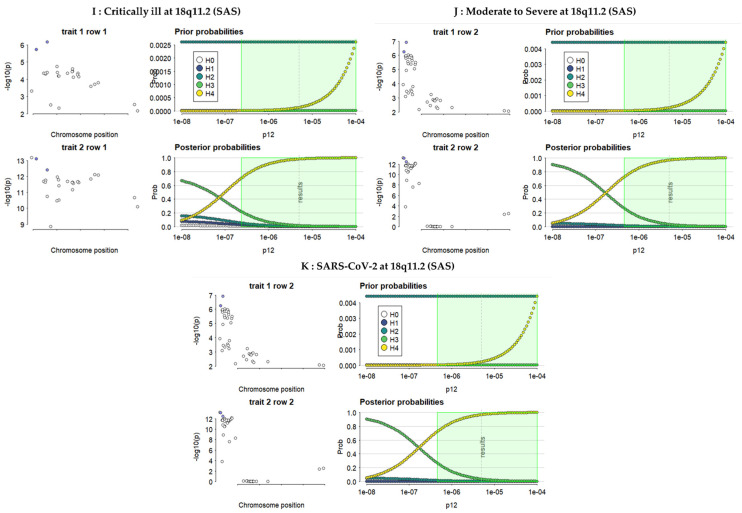
Sensitivity analysis and robustness of shared causal signals for both COVID-19 and CAD under the assumption that H3 and H4 are approximately equal. The left panels display local Manhattan plots of the GWAS summary statistics, displaying −log10 *p*-values for the 1p31.1, 8p21.3, and 18q11.2 loci with fine mapped signals in purple dots. The right panels show the prior and posterior probabilities for hypotheses *H*_0_ through *H*_4_ as a function of the prior probability of colocalization (*P*_12_ < 5×10^−6^). A vertical line indicates the threshold used for sensitivity checks. Blue and green points highlight SNPs in strong linkage disequilibrium (*r*^2^ > 0.8) with the prioritized causal variants. Panels (**A**–**G**) show the allelic effects of the lead SNPs on COVID-19 and CAD using the EUR data, while panels (**H**–**K**) provide the corresponding effects using SAS data from the 1000 Genomes Project.

In the EUR population, fine mapping prioritized rs2133204 at the 1p31.1 locus as the primary signal for CAD. This signal overlapped with the lead signal rs7515509 for COVID-19 in critically ill and moderate to severe hospitalized patient groups (*PPH*_4(abf)_ = 0.70 and 0.94, respectively; Table 2; Figure 2A,B). A strong pairwise linkage disequilibrium (D′ = 0.97, *p* < ×10^−4^) confirmed that these overlapping variants are genetically linked (Figure S4A; Table S4).

At the 8p21.3 locus, the signal rs8192327 for the critically ill group colocalized with the CAD signal rs56390102 (*PPH*_4(abf)_ = 0.70; D′ = 0.64, *p* < ×10^−4^), and the lead signal rs8192330 for the moderate to severe hospitalized COVID-19 group colocalized with CAD signal rs56408342 (*PPH*_4(abf)_ = 0.85; D′ = 0.98, *p* < ×10^−4^; Table 2; Figure 2C,D; Figure S4B, Table S4). Notably, no significant colocalization was observed for the general SARS-CoV-2 group at both 1p31.1 and 8p21.3 loci.

At 18q11.2, we observed consistent evidence of colocalization across all COVID-19 phenotypes. The lead variant rs4800403 from the critically ill COVID-19 group colocalized with CAD signal rs16967171 (*PPH*_4(abf)_ = 0.98; D′ = 0.98, *p* < ×10^−4^; Table 2; Figure 2E; Figure S4C, Table S4), while the same variant rs4800403 in the moderate to severe hospitalized COVID group overlapped with CAD variant rs3813126 (*PPH*_4(abf)_ = 0.93; D′ = 0.98, *p* < ×10^−4^; Table 2; Figure 2F; Figure S4C, Table S4). Furthermore, the general SARS-CoV-2 group showed strong evidence of colocalization between rs16967171 and CAD (D′ = 1.0, *p* < ×10^−4^; *PPH*_4(abf)_ = 0.99) (Table 2; Figure 2G; Figure S4C, Table S4).

Pleiotropic signals were fine mapped through the Coloc Bayesian framework, integrated with the Sum of Single Effects (SuSiE) regression model at the colocalized loci (1p31.1, 8p21.3, and 18q11.2). LD metrices were constructed from the 1000 Genomes Project (1KGP) for the EUR and SAS populations. The analysis prioritized causal signals for both traits across the three COVID-19 phenotype groups. Colocalization evidence is quantified using posterior probabilities (*PP*) derived from Approximate Bayes Factors (ABF) as described in the methods below.

In the SAS ancestry, including the Lahore Punjabi population in Pakistan, we identified a shared genetic signal rs56390102 from the critically ill COVID-19 group and CAD at the 8p21.3 locus, with strong evidence of colocalization (*PPH*_4(abf)_ = 0.74; D′ = 1.0, *p* < 1 × 10^−4^; Table 2; Figure 2H; Figure S4D; Table S4). However, no significant colocalization was observed at this locus for either the moderate to severe hospitalized or the general SARS-CoV-2 groups.

At the 18q11.2 locus, the lead signal rs4800403 from both the critically ill and the moderate to severe hospitalized phenotypes overlapped with CAD signals rs16967171 and rs12958355, respectively, with high confidence (*PPH*_4(abf)_ = 0.98 and 0.74, respectively; D′ = 0.92, *p* < ×10^−4^; Table 2; Figure 2I,J; Figure S4E and Table S4). In the general SARS-CoV-2 group, rs16967171 colocalized with the CAD signal rs12958355 (*PPH*_4(abf)_ = 0.99; D′ = 0.92, *p* < ×10^−4^; Table 2; Figure 2K; Figure S4E and Table S4). No evidence of colocalization between any COVID-19 group and CAD was detected at the 1p31.1 locus for the moderate to severe hospitalized group and at the 8p21.3 locus for the general SARS-CoV-2 groups.

In the European samples, the lead signal rs7515509 showed strong linkage disequilibrium with neighboring associated signals at the 1p31.1 locus (Figure S4A; Table S4). At the 8p21.3 locus, the lead signal rs8192330 showed high genetic linkage with all identified signals from both the COVID-19 and CAD cohorts. (Figure S4B and Table S4). At the 18q11.2 locus, the lead signal showed significant linkage disequilibrium with proxy (secondary) signals associated with either COVID or CAD (Figure S4C and Table S4). For the SAS population, a similar LD pattern was observed for all prioritized signals for both COVID and CAD (Figure S4D and Table S4).

### 2.5. Causal Association Between COVID-19 and CAD

#### 2.5.1. Forward Mendelian Randomization (MR) Analysis

**Figure 3:**
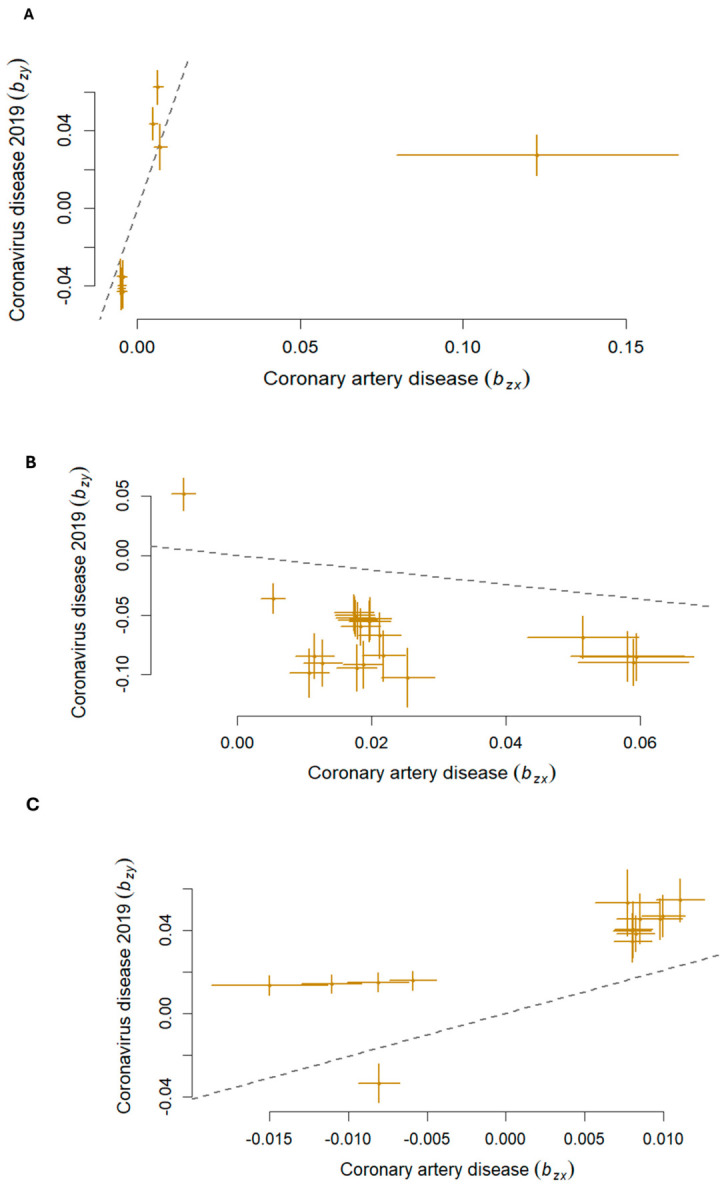
Scatter plots showing the genetic effect of a leading SNP associated with CAD on the exposure to COVID-19 and vice versa determined through bidirectional GSMR at the three pleiotropic loci (**A**–**C**). The X-axis represents the effect size from CAD. The Y-axis represents the effect size from COVID-19 The dash line denotes the regression slop, while horizontal and vertical lines represent the effect size of each variant.

Forward GSMR analysis with curated GWAS datasets revealed a statistically significant causal effect of CAD on COVID-19 risk across three genomic regions (Figure 3; Table 3). Significant causal estimates were observed at 1p31.1 ($\hat{\beta }$_xy_ = 4.92, *P*_Adj(forward-GSMR)_ < 4.21 × 10^−3^), 8p21.3 ($\hat{\beta }$_xy_ = −0.61, *P*_Adj(forward-GSMR)_ < 3.19 × 10^−2^), and 18q11.2 ($\hat{\beta }$_xy_ = 2.07, *P*_Adj(forward-GSMR)_ < 2.30 × 10^−6^). The HEIDI (Heterogeneity in Dependent Instruments) outliers, the genetic variants that violate a core assumption of MR, identified during the GSMR analysis, including rs1909203, rs6995980, rs116825679, rs73225858, rs177990, rs579332, rs678308, and rs542228, were excluded to ensure that the instrumental variables would provide a less biased and more reliable estimate on the direct causal relationship between CAD and COVID-19.

#### 2.5.2. Reverse MR Analysis

Reverse GSMR analysis revealed significant causal effects of COVID-19 on CAD risk at two genetic loci: the 8p21.3 locus ($\hat{\beta }$_z**x**_ = 0.10, *P*_Adj(reverse-GSMR)_ < 2.20 × 10^−4^) and the 18q11.2 locus ($\hat{\beta }$_z**y**_ = 0.15, *P*_Adj(reverse-GSMR)_ < 1.72 × 10^−7^) (Figure 3 and Table 3). In contrast, the 1p31.1 locus (Chr1:77695983–78192445) showed no significant association ($\hat{\beta }$_z**y**_ = 2.4 × 10^−3^, *P*_Adj(reverse-GSMR)_ < 0.83) (Figure 3; Table 3). The HEIDI outliers identified during the reverse GSMR analysis, including rs116825679, rs56408342, rs73225858, rs177990, rs579332, rs678308, and rs542228, were excluded from the analysis.

### 2.6. Likely Causal Genes at the Co-Incident Loci Shared by COVID-19 and CAD

**Figure 4:**
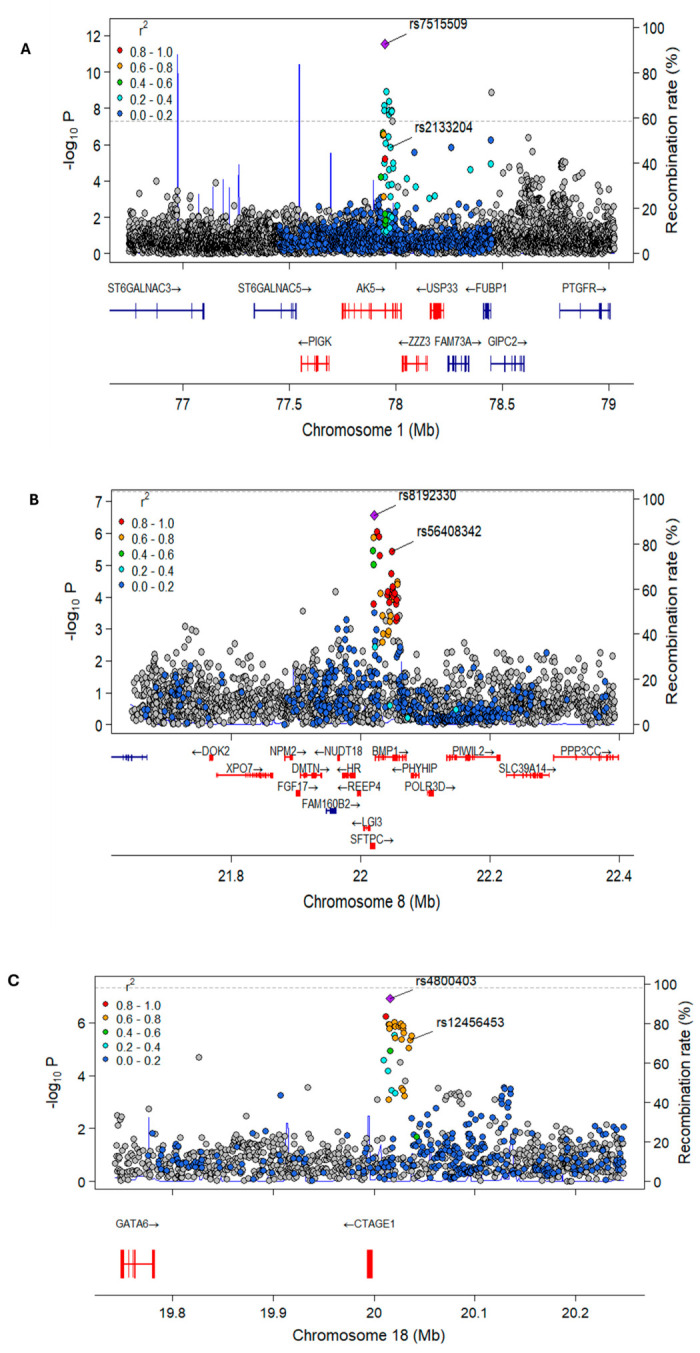
LocusZoomR regional plots (panels **A**–**C**) showing the spatial association of one lead SNP for COVID-19 and one for CAD with likely candidate genes at the three pleiotropic loci (1p31.1), (8p21.3), and (18q11.2) respectively**.** Lead SNPs are denoted in the figures. The red lines denote prioritized genes identified in the gene level analysis with the length of each line corresponding to the physical size of the respective genes.

We applied the mBAT-Combo framework using GENCODE (v.40) for the hg38 reference genome to prioritize candidate genes driving the shared molecular pathology of COVID-19 and CAD across the curated GWAS datasets. At the 1p31.1 locus, we identified four genes (*AK5, PIGK, USP33,* and *ZZZ3*), spatially associated with the lead signal rs7515509 (*p* < 2.94 × 10^−12^; *P*_mBAT-Combo_ < 9.08 × 10^−6^) as likely candidate genes (Figure 4, Table 4). At the 8p21.3 locus, we found eighteen genes (*DMTN, FHIP2B, DOK2, XPO7, NPM2, FGF17, NUDT18, HR, HRURF, REEP4, LGI3, SFTPC, BMP1, PHYHIP, POLR3D, PIWIL2, SLC39A14*, and *PPP3CC*) spatially associated with the lead rs8192330 (*p* < 6.46 × 10^−6^; *P*_mBAT-Combo_ < 2.83 × 10^−7^). And at the 18q11.2 locus, we found *CTAGE1* and *GATA6* as candidate genes spatially associated (*p* < 1.27 × 10^−7^; *P*_mBAT-Combo_ < 1.43 × 10^−6^) with the pleotropic signal rs4800403 (Figure 4, Table 4). LocusZoom plots visualized their genomic location, statistical significance, gene locations, and LD patterns of SNPs simultaneously (Figure 4, Table 4).

### 2.7. Expression Profiling of COVID-19 Cases and Candidate Genes Prioritization

**Figure 5:**
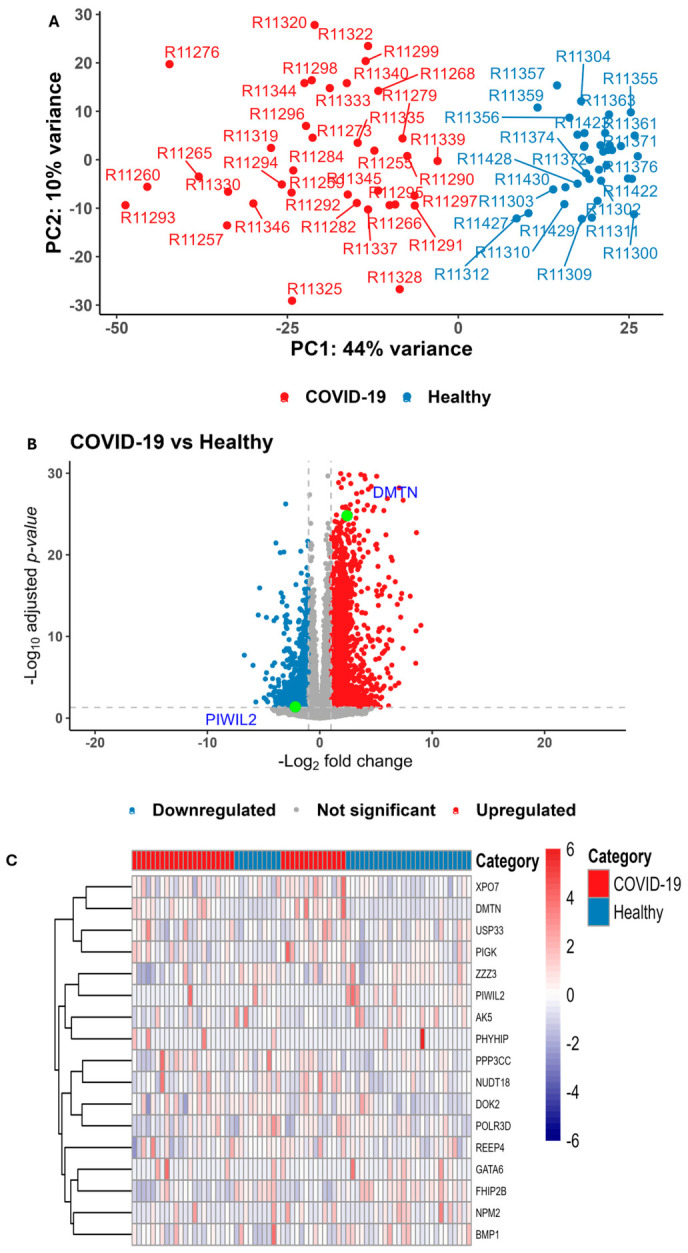
Altered gene expression in peripheral mononucleate blood cells (PBMCs) of patients with COVID-19. (**A**) Principal component analysis (PCA) cluster plot of GSE202805 dataset, illustrating transcriptomic profiles from PBMCs of individuals with COVID-19 (red), and healthy subject (blue). PC1 and PC2 denote the maximum variance in gene expression. (**B**). Volcano plot showing differential gene expression in PBMCs from COVID-19 patients and healthy. The X-axis denotes −log2 fold change, and the Y-axis denotes −log 10 *p*-value. The blue dots denote significantly downregulated genes, and the red dots denote significantly overexpressed genes (*p*-value < 0.05). *DMTN* and *PIWIL2* are labeled with light green dots. (**C**). The heatmap visualizing gene expression patterns across subjects with COVID-19, and healthy. The dendrograms show differentially expressed genes in the lines. In the heatmap, each gene is mapped to a color gradient representing its normalized expression level across the samples (from bright blue = low level of expression to dark red = high level of expression).

Following normalization, principal component analysis (PCA) confirmed the effective sample mitigation and consequently 36 COVID-19 and 37 healthy samples, comprising 60,603 observations (genes) were retained for further analysis (Figure 5A). No batch correction was applied, and no obvious batch-related structure was observed in PCA. DESeq2 identified 3728 differentially expressed genes in PBMCs with log2 fold-change values ranging from −6.72 to 11.34(*P*_(Adj)-DESeq2_ < 0.05; |log2FC| ≥ 1 for up and |log2FC| ≤ −1 for downregulated genes, respectively), (Table S5). Of the significant genes, 1963 genes were upregulated and 1765 were downregulated in COVID-19 patients (Table S5). Cross-matching the differentially expressed genes (Table S5) with our prioritized genes (Figure 4, Table 4) validated *DMTN* and PIWIL2 as the top candidate genes (Table 5, Table S5). *DMTN* (*P*_(Adj)-DESeq2_ < 1.60 × 10^−25^, log_2_FC = 2.42) was upregulated 5.35-fold, and the *PIWIL2* (*P*_(Adj)-DESeq2_ < 4.21 × 10^−2^, log_2_FC = −2.18) was downregulated 4.53-fold in COVID-19 patients (Figure 5B, Table 5) (Table S5).

Expression profiling further validated proxitropy, a phenomenon where a signal variant is associated with the expression of multiple, neighboring genes. We observed proxitropic associations of pleotropic variant rs8192330 (*p* < 2.83 × 10^−7^) with the expression of *DMTN* (*P*_(Adj)-DESeq2_ < 1.60 × 10^−25^) and *PIWIL2* (*P*_(Adj)-DESeq2_ < 4.21 × 10^−2^) at the 8p21.3 locus (Figure 5B; Table 5), and the variant rs7515509 (*p* < 2.94 × 10^−12^) with the expression of PIGK (*P*_(Adj)-DESeq2_ < 2.23 × 10^−2^) at 1p31.1 (Table S5).

#### 2.7.1. Hierarchical Clustering

We observed that clear transcriptomic separation between healthy and COVID-19 samples was not evident, except when analyzed using clustering_distance_cols = “binary”. The dendrogram (Figure 5C) reveals how genes are hierarchically clustered based on the similarity of their expression patterns across all samples. Genes that are “closer” together on the tree branches have more similar expression levels between the two clinical groups of subjects. Heatmap shows *DMTN* is upregulated and *PIWIL2* is down-expressed in COVID-19 as compared to healthy.

#### 2.7.2. Gene Set Enrichment Analysis and Pathways Altered by COVID-19 Infection

**Figure 6:**
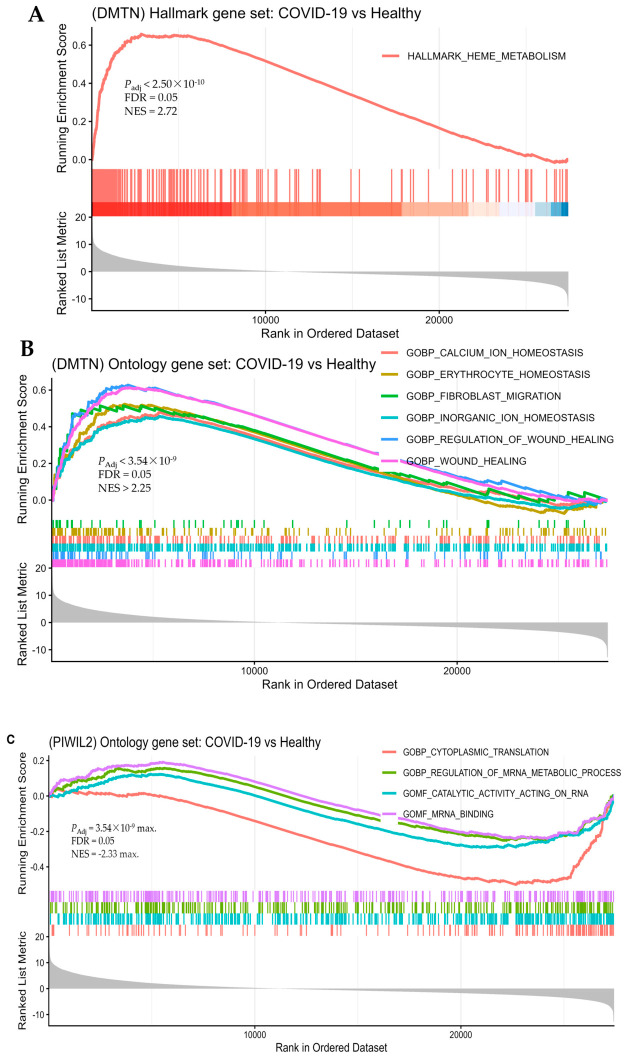
Gene set enrichment analysis of differentially expressed genes in peripheral mononucleate blood cells of patients with COVID-19 versus healthy. (**A**) Hallmark of heme metabolism from hallmark gene sets enrichment analysis with DMTN over expression by GSEA. The color band in the bottom represents up (left) to down (right) trend of genes expression in the hallmark (**B**) Overexpressed *DMTN* implicating enriched pathways from Ontology gene sets (**C**). Enriched pathway implicated by lower expression of *PIWIL2* from Ontology gene sets. The color band (**B**,**C**) in the bottom represents down (left) to up (right) trend of genes expression in the respective pathways.

Gene set enrichment analysis (GSEA) of PBMC transcriptomic data showed significantly altered pathways associated with *DMTN* (virtually all upregulated), and *PIWIL2* (virtually all downregulated) in COVID-19 patients compared to healthy (Figure 6A–C; Tables S6 and S8). *DMTN* is involved in several key biological pathways, including heme metabolism, and multiple gene ontology biological processes (GOBPs). These include Fe ^+ +^/Ca ^+ +^ homeostasis, intracellular ion homeostasis, erythrocyte homeostasis, fibroblast migration, wound healing and its regulation, and regulation of cytoskeleton organization (Figure 6A,B; Tables S6 and S7). 

In the upregulated heme metabolism HALLAMARK pathway, the genetic signatures responding to the glutathione pathway (*SLC7A11*, *GCLM*, *NCOA4*, and *EPOR*) (Table S6), and heme biosynthesis (*ALAS2*, *FECH*, and *SLC4A*) (Table S6) were upregulated. Among the enriched GOBPs, Fe ^+ +^ homeostasis, in particular, was prominently featured by the upregulation of key iron regulating genes (*SLC40A1*, *FTH1*) (Table S7). *FTH1* encodes H-ferritin, which is universal protein for intracellular storage, distribution of iron, and important inflammatory markers [32]. Among other upregulated GOBP pathways, associated with *DMTN*, were fibroblast migration, wound healing, and wound healing regulation (Figure 6A,C; Tables S6 and S7). The details of all *DMTN* implicated pathways are shown in Table S7. Similarly, gene set enrichment analysis (GSEA) reported that *PIWIL2* is associated with significantly downregulated biological pathways: Cytoplasmic translation and regulation of mRNA metabolism (Figure 6C).

## 3. Discussion

This study was designed to quantify the genetic connection between COVID-19 and CAD, and the shared genetic architecture and their associated cellular mechanisms linking both traits. In this study, we provide strong evidence for a shared genetic susceptibility between COVID-19 and coronary artery disease (CAD). We observed significant positive genetic correlations both at the whole-genome-wide level and locally at the locus level. One robust aspect of this study is the consistency of the genetic correlation across different severity and European and Southeast Asian (including Lahore Punjabi) populations. Both moderate and severe COVID-19 populations showed an increased susceptibility to CAD. This increased susceptibility has been observed in both European (EUR) and South Asian (SAS) ancestries (Table 1). The finding that the Lahore Punjabi population in Pakistan shared these genetic risk markers suggests that the link between COVID-19 and CAD is not population-specific but rather a global biological phenomenon. While the magnitude of association was low, the level of statistical significance across all three groups reinforces the validity of the genetic overlap due to enough power behind the large genome. The observed global genetic correlation between CAD and COVID-19 was low, at a range of 0.10–0.15, though statistically significant. The large sample size provided sufficient statistical power to detect even nominal local relationships. A recent review found that many patients who recovered from COVID-19 continued to experience complications, including CHD, even without detectable viral infection [29]. The clinical implications confirm individuals at “double risk” for both severe viral outcomes and heart disease. Although a few studies have demonstrated that COVID-19 significantly increases the risk of CAD and major adverse cardiac events for up to two to three years following the acute infection [4,33,34], this study did not include a longitudinal post-COVID cohort, incident CAD follow-up, adjusted hazard ratios, confidence intervals, or epidemiologic control for confounding and surveillance bias. Systemic inflammation and endothelial dysfunction serve as the shared pathophysiological mechanisms underlying these outcomes [11,12,13]. These results align with previous studies, with minor variations likely due to differences in methodology and datasets [14,35]. In this study, we identified 24 coincident risk loci for COVID-19 and CAD (Table S1). Our results are consistent with Guo et al., where the PLEIO approach for multiple-trait analysis was used to detect 10 shared loci between COVID-19 and CAD [36,37]. The use of HyPrColoc and fine mapping (SuSiE) allowed us to prioritize specific genetic signals that bridge the two disorders. Many genomic regions in colocalization show mere physical “coincidence”, harboring separate causal variants for each disease. This suggests that while these genomic linkages are important to both diseases, the functional effects are largely independent (Table S1). This study identified 3 critical pleiotropic loci at 1p31.1, 8p21.3, and 18q11.2 where specific genetic variants drive risk for both conditions simultaneously (Table 2; Figure 2; Tables S2 and S4 and Figure S4A–D). We found high confidence evidence (*PP*_HyPrColoc_ > 0.75) that single causal variants influence both traits (Table S2), as described in this study [38]. These represent the most direct biological links between acute viral severity and chronic cardiovascular disease. The high linkage disequilibrium at locus 1p31.1 suggests the variants are inherited together, creating a unified risk block for moderate to severe COVID-19 and CAD (Table 2; Figure 2; Figure S4A–D, Table S4). Pietzner et al. used HyPrColoc to identify the genetic architecture of host proteins involved in SARS-CoV-2 infection [39].

The 8p21.3 locus showed a strong negative genetic correlation across both European and South Asian ancestries. This suggests that while the locus is shared, the genetic directionality of risk at this specific site may act differently across phenotypes. The 18q11.2 locus exhibited the strongest correlation in South Asian ancestry (Table 1). Positional candidate gene *GATA6* (Table 4) is a transcription factor vital for heart and lung repair and is a potential shared driver of both severe pneumonia and coronary damage. Previous studies have demonstrated the involvement of *GATA6* in both disorders [40,41].

Our Mendelian Randomization analysis provides critical insights into the causal relationship between COVID-19 and CAD. The forward Mendelian Randomization analysis established that a genetic predisposition to CAD significantly increases the risk of COVID-19 at three specific loci (Figure 3; Table 3). This finding suggests that the physiological state or pathways associated with CAD such as chronic inflammation or vascular fragility may create a primed environment for severe viral infection. The reverse Mendelian Randomization analysis explored whether the genetic signals for COVID-19 contribute to the development of CAD (Figure 3; Table 3). The 1p31.1 locus showed no significant reverse causality, suggesting that while CAD can influence COVID-19, the reverse is not true. These findings provide a genetic explanation for the clinical observation that heart disease and COVID-19 are inextricably linked. The bidirectional causality at 18q11.2 and 8p21.3 suggests a feedback loop where cardiovascular vulnerability worsens viral infection, and the resulting viral infection, in turn, accelerates CAD. These results highlight the necessity of long-term cardiovascular monitoring for COVID-19 survivors, particularly those with a genetic predisposition involving these specific loci. Epidemiological and experimental evidence indicate COVID-19 infection significantly increases the risk of major adverse cardiac and thrombotic events [5,6,7,8,9,10]. Conversely, pre-existing obesity, heart failure, and ischemic heart disease are major risk factors for increased susceptibility, severity of coronavirus infection, and the development of long COVID-19 syndrome [4].

The integration of the mBAT-Combo framework with transcriptomic data from peripheral blood mononuclear cells (PBMCs) prioritized a list of candidate genes that likely drive the shared pathology of COVID-19 and CAD (Figure 4; Table 4 and Table 5, Table S5). Four genes were identified at 1p31.1 (rs7515509) locus, including *AK5*, *PIGK*, *USP33*, and *ZZZ3*. The association of *PIGK* is particularly notable as it was validated through expression profiling (Table S5). At the 8p21.3 locus (rs8192330), 18 candidate genes were identified, including *DMTN*, *PIWIL2*, *FHIP2B*, *DOK2*, and *SFTPC* (Figure 5; Table 5; Table S5). The high number of genes at this site suggests a complex regulatory environment where multiple biological pathways may be affected. For the 18q11.2 locus (rs4800403), the transcription factors *GATA6* and *CTAGE1* were prioritized (Table 4). Given *GATA6*’s role in cardiovascular and pulmonary development, it remains a primary candidate for mediating tissue repair in both diseases. Our spatial findings align with previous studies; for example, variants associated with diabetes and obesity include 86 intronic variants of *FTO* [42], with the lead SNP (rs9930506) showing spatial associations that affect *IRX3* gene regulation. Similarly, SNPs (rs1297265, rs1736020, and rs2823286) in an intergenic region of chromosome 21 are associated with the distant *NRIP1* gene rather than the closer *USP25* gene [43].

The integrated analysis of transcriptomic profiling and gene set enrichment revealed how those shared variants drive the systemic damage observed in both severe COVID-19 and CAD. The functional analysis confirms that *DMTN* and *PIWIL2* are not just coincidentally located near risk variants (Table 4 and Table 5, Table S5); they are actively dysregulated during COVID-19 infection. The shared genetic architecture between CAD and COVID-19 likely manifests as a dual burden: a predisposition to iron dysregulation and oxidative stress (*DMTN*) and a compromised pulmonary/vascular defense (*PIWIL2*). These findings provide a biological roadmap for understanding why some patients are genetically “primed” for both severe viral outcomes and long-term coronary damage. Defects in iron homeostasis, dysregulated erythropoiesis and immune dysfunction due to COVID-19 possibly contribute to inefficient oxygen transport, inflammatory disequilibrium and persisting symptomatology, and therapeutically tractable [44]. The pathological role of iron and associated oxidative stress equally play role in CAD [45,46,47,48]. Previous studies indicated that SARS-CoV-2 severely disrupts mRNA metabolism, including tRNA aminoacylation and miRNA pathways, which facilitates the aberrant immune response seen in critical patients. By manipulating host machinery for replication, the virus induces high-level cytokine production and impairs interferon signaling. This leads to significant metabolic shifts, such as mitochondrial dysfunction in immune cells. Furthermore, hypoaccurate translation within the mitochondria results in increased oxidative stress, which is sufficient to influence protein synthesis. This process of translation remodeling can be mediated by reversible changes in the redox state of protein synthesis components susceptible to oxidation. In the broader context of cardiovascular pathology, such as coronary artery disease (CAD), cytoplasmic translation plays a heavy role in the cellular response to ischemia, stress, and remodeling [49,50,51,52,53,54,55].

## 4. Materials and Method

### 4.1. Datasets and Study Populations

Meta-analysis GWAS summary statistics for COVID-19 were obtained from the COVID-19 Host Genetic Consortium (HGI) (https://www.covid19hg.org/, accessed on 28 April 2026) release round 7, focusing on three predefined phenotypes: (1) Critically ill cases (18152 subjects requiring respiratory support in hospital or who died), (2) Moderate to severe cases (44,986 hospitalized subjects), and (3) SARS-CoV-2 reported cases (159,840 cases and over 6 million controls across 64 studies) [25,26,56,57,58]. The first two categories represent the severity of COVID-19, while the third reflects COVID-19 susceptibility [59].

Corresponding GWAS summary datasets for CAD were obtained from the Coronary ARtery DIsease Genome Wide Replication and Meta-analysis plus The Coronary Artery Disease Genetics consortium (CARDIoGRAMplusC4D) (https://www.cardiogramplusc4d.org/, accessed on 28 April 2026), encompassing 22,233 cases and 64,762 controls in CARDIoGRAM and 15,420 CAD cases and 15,062 controls in C4D GWAS [60]. CAD participants were predominantly of European (EUR) ancestry, while the COVID-19 subjects were of different genetic ancestries, including admixed American, African, East Asian, European, Middle Eastern, and South Asian participants. To prioritize shared causal variants for both traits, we performed fine mapping utilizing LD matrices derived primarily from European ancestry. To improve the resolution of these signals and account for global genetic diversity, we included a South Asian population, specifically the Lahore Punjabi (PJL) population in Pakistan. RNA-seq expression profiling (GSE202805) of peripheral blood mononuclear cells (PBMCs) from COVID-19 patients was retrieved through the Gene Expression Omnibus repository [61]. These data were generated using the Illumina HiSeq 4000 high-throughput sequencing platform. The cohort includes healthy (*n* = 10), acute-mild (*n* = 4), acute-moderate (*n* = 6), acute-severe (*n* = 32), and convalescent (*n*= 28) subjects. We compared “COVID-19” samples (*n* = 42) versus “healthy” (*n* = 38) as described in Section 4.3.

### 4.2. Post-GWAS Analyses

We conducted post-GWAS analyses for both diseases, as outlined in Figure S1. The obtained COVID-19 dataset included 14,415,897 distinct single-nucleotide polymorphisms (SNPs) from three predefined categories, and the CAD dataset included 20,853,377 unique SNPs from GWAS summary datasets for CAD. We cross-referenced these datasets to identify shared biallelic SNPs. Rare variants, defined as those reported to have allele frequencies of less than 1% in either GWAS, were excluded. This resulted in a set of 7685,407 identical SNPs for both traits referred to as the GWAS-curated datasets. For high-coverage whole-genome sequencing samples from the 1000 Genomes Project (1KGP) resource, we applied standard data quality control procedure using PLINK2 [62,63,64]. Quality control was performed by applying the following filters: missing rate per variant (–geno 0.2), missing rate per individual (–mind 0.2), minor allele frequency (–maf 0.01), and Hardy–Weinberg equilibrium (–hwe midp). The quality controlled 1KGP genotypic data were used to extract biallelic European and South Asian samples, including the Lahore Punjabi population in Pakistan and named it 1KGP-curated data, which served as a reference panel for constructing LD matrices for respective populations.

#### 4.2.1. Evaluation of Global Genetic Connections Between COVID-19 and CAD

We assessed the genetic correlation and covariance across the genome between three predefined COVID-19 phenotypes and CAD using the LD score regression model in the R implementation of ldsc software (version 1.0.1) [65,66]. In the model, we estimated LD scores for SNPS present in both GWAS-curated datasets in a customized way using LD matrices on the EUR and SAS samples (1KGP-curated data), with a window size of 1 centimorgan (cM). The computed LD scores were, then, used to predict the genome-wide genetic associations between CAD and three COVID-19 phenotypes from HGI.

#### 4.2.2. Functional Genomic Coordinates or Genomic Risk Loci

We defined functional genomic regions, or risk loci, using index SNPs in the GWAS-curated datasets (*4.2.0 Post-GWAS analyses*) by PLINK (https://www.cog-genomics.org/plink/2.0/, accessed on 28 April 2026) v.2 [64,67]. The genome-wide significant SNPs (*p* < 5 × 10^−8^) were clumped at an *r*^2^ threshold ( < 0.1) to identify independent lead signals within each risk locus. We assigned a window ( ± 250 kb) centered on each lead signal to serve as the functional block for each locus [68]. Biallelic variants grouped with lead signals at pairwise *r*^2^ (0.1 ≤ *r*^2^ < 0.6), or SNPs within the window, were assigned a *p*-value threshold of ≤ 0.1 to ensure robust results and minimize noise in downstream analyses such as colocalization and fine mapping. The lead signal with the smallest *p*-value was designated as the top signal, while all others within the locus were considered secondary signals. We used a GRCh38 1KGP reference panel to establish risk loci through pairwise *r*^2^. When multiple significant signals were present in a genomic interval, we separated them using haploReg LD data with the haploR v.4.2 package to determine if the region harbors more than one causal or independent variants [69,70].

#### 4.2.3. Trait–Trait Colocalization

Finding shared genetic links (overlap and pleiotropy) between COVID-19 and CAD was achieved through the horizontal integration of summary statistics from two separate GWAS. We used COVID-19 and CAD GWAS-curated datasets for colocalization and employed three models for defining genetic colocalization, namely, Approximate Bayes Factor (ABF) from Coloc v.5.2.3 [31], Hypothesis Prioritization for multi-trait Colocalization from HyPrColoc v.0.0.2 [71], and comparison of locus plots from LocusCompareR v.1.0.0 [72].

ABF identifies either (1) the regions having pleiotropic effect on both traits through single common casual or (2) common regions shared by both traits for their causality but with distinct casual variants based on combined posterior probability [*PP*(*H_3_* + *H_4_*)] with a high degree of confidence (*PP* > 0.7). This indicates that COVID-19 and CAD share genomic regions on chromosomes, either with distinct causal variants [*H*_3_] or with common causal signals [*H*_4_], with high confidence. Specifically, *H*_3_ indicates that both traits are associated with distinct causal variants within the same region, whereas *H*_4_ suggests a single shared causal variant. We assigned genetic signals in each risk locus from the curated GWAS data with default prior parameters *P_1_* = *P_2_* = 1 × 10^−4^ for the prior probability that a SNP was causally associated with trait 1 or trait 2. The prior probability, $P_{12}$, that a SNP is associated with both traits was set to be 5 ×  [31,73]. To identify the colocalized loci containing a single causative SNP with a pleiotropic effect on both traits, we implemented the R instance of HyPrColoc v.0.0.2 [71], which is a Bayesian divisive clustering algorithm using GWAS-curated datasets as input. The loci were considered pleiotropic if *PP*_HyPrColoc_ ≥ 0.75, as indicated by Fotios Koskeridis et. al. [38]. Genetic loci that met the specified colocalization criteria [*PP*_HyPrColoc_ ≥ 0.75 and *PP*(*H*_3_ + *H*_4_) ≥ 0.7] were further visually inspected using LocusCompare plots. A Venn diagram was used to summarize the results of different colocalization methods. The shared loci identified from colocalization have 99% chances to have a (individual or genetically linked) causal signals for the loci and we treated them as 99% credible set of consensus loci for overlapping casual signals. A total of 99% credible sets were derived from coloc.abf for colocalized loci at a predefined rule of posterior *P*(*H*4) > 0.7.

#### 4.2.4. Evaluation of Local Genetic Connections Between COVID-19 and CAD

To assess the genetic correlation and covariance of the genomic regions or the loci identified from Section 4.2.3. Trait–Trait Colocalization, the COVID-19 phenotype showing the highest correlation was recruited along with CAD. The LD score regression as described in Section 4.2.1. was performed for the colocalized loci. We estimated LD scores for each colocalized loci using LD matrices on EUR and SAS samples, with a window size of 1 centimorgan (cM) and applied in the model to predict the genetic correlation at the locus level between COVID-19 and CAD.

#### 4.2.5. Fine Mapping and Prioritizing Pleotropic Variants

Fine mapping aims to detect joint-association signals for causal inference, where the strength of joint association is assessed using the posterior inclusion probability (PIP). We performed fine mapping using the Coloc R package, which incorporates the Sum of Single Effects (SuSiE) model [74,75] to more accurately pinpoint potential causal variants [76,77]. In the analysis, the colocalized loci from trait–trait colocalization were analyzed for the shared casual signals utilizing meta-analysis summary data from CAD along with three COVID-19 phenotypes. LD matrices required in the analysis were constructed from European and South Asian 1KGP-curated data using PLINK2 [64,67]. The posterior probability (*PPH*_4_) from the SuSiE model in the Coloc served as posterior probability for colocalization in SNP-level metric called posterior inclusion probability, which shows that a specific individual overlapping signal is the causal one for each of the three loci, and we considered a high degree of confidence (*PPH*_4(abf)_ ≥ 0.7). For the proxy variants where the lead SNPs were fine mapped with the proxy variants from the respective loci, we considered the identified lead SNPs as defined in Section 4.2.2. Functional Genomic Coordinates or Genomic Risk Loci from each locus as pleotropic for both traits, by analyzing the linkage disequilibrium (LD) patterns of colocalized proxy variants (secondary variants) with the lead one in both ancestries. The SuSiE model requires an estimate of the number of independent causal signals within a locus as an input parameter. Within each identified genomic region, we fitted the Coloc based SuSiE summary statistics model with 100 iterations, setting the expected number of pleotropic signals to match the number identified loci.

#### 4.2.6. Bidirectional Mendelian Randomization (MR)

We applied Generalized Summary data-based Mendelian randomization (GSMR) to assess the causal relationship between COVID-19 and CAD using publicly available GWAS summary data [78,79]. Lead pleiotropic SNPs associated with either COVID-19 or CAD (*p* < 5 × 10^–8^), along with independent signals (*p* < 5 × 10^–3^ and *r*^2^ < 0.05), served as instrumental variables (IVs) in forward and reverse MR analyses, respectively. Colocalized loci underwent LD pruning (*r*^2^ < 0.05 between lead and independent signals; *p* < 5 × 10^–3^) within a 500 kb window using PLINK2, keeping 1KGP-curated data as a reference [64,67]. In bidirectional analysis using GSMR2 v. v1.1. [78,79], we applied a GWAS *p*-value threshold (*p* < 5 × 10^–3^) to select SNPs, an False Discovery Rate (FDR) threshold (≤ 0.05) to exclude chance correlations, and a multi-SNP-based Heterogeneity in Dependent Instruments (HEIDI)-outlier threshold (*p* < 0.01) to remove SNPs that influence both the exposure and the outcome traits through independent pathways in the global HEIDI-outlier method [80]. We also conducted univariate MR analyses for each instrumental variable and plotted the association strength against the causal estimate ($\hat{\beta }$) for all exposure-outcome pairs.

#### 4.2.7. Gene-Level Analysis

mBAT-combo is a novel set-based statistical test designed for gene-based association analysis in post-GWAS research that offers improved power over existing methods [81]. Unlike traditional single-SNP analyses that test one genetic marker at a time, mBAT-combo aggregates the association signals of multiple SNPs within predefined sets, typically defined by gene boundaries. This approach combines Multivariate set-Based Association Test (mBAT) and Fast set-Based Association Test (fastBAT) statistics using a Cauchy combination method. We utilized mBAT-combo v.0.0.0.9 [81] to prioritize the genes most likely involved in the shared risks of COVID-19 and CAD for each pleiotropic locus identified through trait–trait colocalization. We used GWAS-curated datasets for COVID-19 and CAD to prioritize the genes and utilized the mixed ancestry linkage disequilibrium reference panel comprised 3202 samples from the 1KGP. We included protein-coding genes with unique Ensembl gene IDs from GENCODE (v.40) for hg38 (https://www.gencodegenes.org/) to set boundaries. Gene regions were defined as spanning 50 kb upstream and downstream of each gene’s untranslated regions. Predicted genes through mBAT-Combo for both traits were visualized in plots using LocusZoom v.0.3.8 [82] and LDlinkR v.1.4.0 [83] to inspect the genetic architecture, including the recombination rates and linkage disequilibrium patterns among pleiotropic variants.

### 4.3. RNA-Seq Analysis and Candidate Gene Prioritization

In the analysis, RNA-seq expression profiles from 80 peripheral blood mononuclear cells (PBMCs) generated from the Illumina HiSeq 4000 platform were retrieved from the Gene Expression Omnibus (GEO accession #: GSE202805) [61]. The RNA-seq comparison groups were defined in the sample metadata provided in the GEO dataset. Specifically, we combined all acute COVID-19 categories—acute-mild (*n* = 4), acute-moderate (*n* = 6), and acute-severe (*n* = 32)—into a single group, referred to as “COVID-19” (total *n* = 42) and the convalescent (*n*= 28) into another referred to as “healthy” (total *n* = 38) to establish 1:1 ration between two groups. Differential expressions were performed, using the R instance of Deseq2 v.1.48.2 [84], by comparing COVID-19 (*n* = 42) with healthy (*n* = 38). The raw, non-normalized gene count matrices downloaded directly from the GEO dataset were used for the analysis. In the analysis, the differentially expressed genes were, then, compared and cross-matched with genes prioritized from gene-level analysis, which validated candidate genes identified in the preceding gene-level association. DESeqDataSet object was created from the count matrices and conducted normalization, dispersion estimation, and negative binomial modeling procedures [84]. In the procedures, we specified the reference level in the model and pre-filtered genes, retaining only those with at least five counts in a minimum of five samples and genes with low expression across all samples were excluded. The filtered dataset, then, was normalized, estimated for dispersions (a critical step when dealing with relatively small sample sizes), and fitted to the negative binomial models as implemented in DESeq2. We applied variance stabilizing transformation (VST) [85,86,87] with the default blind setting for visualization and other multivariate analyses where raw count data were inappropriate. Log-transformed, normalized expression data of the 500 most variable genes were used for unsupervised principal component analysis (PCA) to identify unintended sources of variation, such as covariates and batch effects and the samples from both groups (COVID-19 and healthy) intermixing the clustering in PCA were excluded in the latter analysis. To minimize noise from low-count genes and improve the quality of gene ranking and visualization in an MA plot, the apeglm package v.1.32.0 for log2 fold change (log2FC) shrinkage instead of the default DESeq2 “normal” estimator was used for analysis [88]. Genes with log2FC ≥ 0.5 and Benjamini–Hochberg (FDR) adjusted *p* < 0.05 were considered significant in expression between groups. These differentially expressed genes were compared with the likely causal genes identified from genome-wide association analyses and the top candidates were visualized using Volcano plots (ggplot2 v.3.5.2) [89]. We considered top candidate genes for gene set enrichment analysis (FGSEA) to predict the altered pathways in COVID-19 patients. The top genes and associated DESeq2 significant signatures from gene set enrichment analysis were exhibited in hierarchical clustering (pheatmap v.1.0.13) [90]. After performing hierarchical clustering of the samples using pheatmap with different distance metrics.

### 4.4. Gene Set Enrichment Analysis and Identification of Altered Pathways from COVID-19 Patients

Gene set enrichment analysis (GSEA) using GSEA function of the clusterProfiler R package (version 4.18.4) [91,92,93,94,95] was implemented using the ranked gene list derived from the DESeq2 results, where genes were ordered based on the *stat* column. We used the default gene set size parameters in GSEA, with a maximum gene set size of 500. For pathway annotations, gene sets were obtained from Molecular Signatures Database (MSigDB) [96] using the *msigdbr* R package (version 26.1.0) [97,98]. Multiple testing correction for pathway enrichment was performed using the Benjamini–Hochberg method [99], and adjusted *p*-values (FDR) were used to determine the significance. The altered biological pathways underlying differentially expressed top candidate genes in COVID-19 patients by referencing three genes sets from MSigDB. These gene sets are hallmark gene sets (H) [97], Ontology gene sets (C5) that include gene ontology (GO) [100], and human phenotype ontology (HPO) [101]. The significantly altered pathways from GSEA for top candidate genes were visualized using the clusterProfiler v. 4.18.4 [91,93,94,95].

## 5. Conclusions

In conclusion, we conducted post-GWAS analysis to identify the shared molecular signatures and their mechanisms linking both traits. This study identifies a shared genetic susceptibility between COVID-19 and coronary artery disease (CAD) across different populations, revealing a “double risk” driven by shared mechanisms such as inflammation and endothelial dysfunction. The analysis identifies 24 shared risk loci and 3 critical pleiotropic loci (1p31.1, 8p21.3, 18q11.2) where single variants drive risk for both conditions, driven by candidate genes like GATA6 that mediate cardiovascular and pulmonary damage. The shared genetic architecture between CAD and COVID-19 likely manifests as a dual burden: a predisposition to iron dysregulation and oxidative stress (*DMTN*) and a compromised pulmonary/vascular defense (*PIWIL2*). These findings provide a biological roadmap for understanding why some patients are genetically “primed” for both severe viral outcomes and long-term coronary damage.

## Figures and Tables

**Table 1:** Genetic correlations between COVID-19 phenotypes and CAD in EUR and SAS populations.

Global Genetic Correlation Between COVID-19 and CAD for European Ancestry								
Phenotype 1 (COVID-19)	Phenotype 2 (CAD)		*R* ^G^	*SE* ^G^	*COV* ^G^	*SE* ^COV^	*Z* score	*P*_R_^G^
Critically ill	Coronary artery disease		1.02 × 10^−1^	1.98 × 10^−2^	4 × 10^−4^	6.98 × 10^−5^	5.19	*p* < 2.03 × 10^−7^
Moderate to severe hospitalized			1.51 × 10^−1^	1.79 × 10^−2^	6 × 10^−4^	7.03 × 10^−5^	8.45	*p* < 2.67 × 10^−17^
SARS-CoV-2 reported cases			1.21 × 10^−1^	1.21 × 10^−1^	4 × 10^−4^	6.72 × 10^−5^	6.60	*p* < 4.04 × 10^−11^
**Global genetic correlation between COVID-19 and CAD for South Asian ancestry including Lahore Punjabi population in Pakistan**								
Critically ill	Coronary artery disease		9.38 × 10^−2^	2.33 × 10^−2^	4 × 10^−4^	1.0 × 10^−4^	4.02	*p* < 5.70 × 10^−5^
Moderate to severe hospitalized			1.57 × 10^−1^	2.32 × 10^−2^	8 × 10^−4^	1.0 × 10^−4^	6.79	*p* < 1.07 × 10^−11^
SARS-CoV-2 reported cases			1.34 × 10^−1^	2.44 × 10^−2^	6 × 10^−4^	1.0 × 10^−4^	5.50	*p* < 3.68 × 10^−8^
**Local genetic correlation between COVID-19 and CAD for European ancestry**								
Phenotype 1 (COVID-19)	Phenotype 2 (CAD)	Coordinate/Locus	*R* ^G^	*SE* ^G^	*COV* ^G^	*SE* ^COV^	*Z* score	*P*_R_^G^
Moderate to severe hospitalized	Coronary artery disease	Chr1:77695983_78192445 1p31.1 (0.49 Mb)	6.58 × 10^−1^	0.17	1.17 × 10^−2^	5.5 × 10^−3^	3.76	*p* < 2.0 × 10^−4^
		Chr8:21773384_22270797 8p21.3 (0.49 Mb)	−8.11 × 10^−1^	0.17	−2.8 × 10^−2^	7.4 × 10^−3^	−4.54	*p* < 5.45 × 10^−6^
		Chr18:19748905_20248798 18q11.2 (0.49 Mb)	4.76 × 10^−1^	−0.12	3.9 × 10^−2^	−1.25 × 10^−2^	3.94	*p* < 7.87 × 10^−5^
**Local genetic correlation between COVID-19 and CAD for South Asian ancestry including Lahore Punjabi population in Pakistan**								
Moderate to severe hospitalized	Coronary artery disease	Chr8:21773384_22270797 8p21.3 (0.49 Mb)	−8.78 × 10^−1^	0.16	−4.04 × 10^−2^	1.16 × 10^−2^	−5.39	*p* < 6.92 × 10^−8^
		Chr18:19748905_20248798 18q11.2 (0.49 Mb)	9.22 × 10^−1^	0.20	4.76 × 10^−2^	2.71 × 10^−2^	4.46	*p* < 7.92 × 10^−6^

Global genetic correlation was estimated using genome-wide linkage disequilibrium score regression (LDSC) to quantify the genome-wide shared genetic architecture between the two traits. Conversely, local genetic correlation was assessed for the three identified loci (1p31.1, 8p21.3, and 18q11.2) using an integrated colocalization framework. Both analyses utilized population-specific LD reference panels from the 1000 Genomes Project (1KGP) for European (EUR) and South Asian (SAS) ancestries. *R*^G^ = Genetic correlation; *SE*^G^ = Standard error of *R*^G^; *COV*^G^ = Genetic covariance; *SE*^COV^ = Standard error of *COV*^G^; *P*_R_^G^ = *p*-value of *R*^G^.

**Table 2:** Coincident pleotropic signals between COVID-19 and CAD.

COVID-19 Phenotype	Coordinate/Locus	COVID-19	CAD	*PPH*_0(abf)_	*PPH*_1(abf)_	*PPH*_2(abf)_	*PPH*_3(abf)_	*PPH*_4(abf)_	Ancestry
Critically ill	Chr1:77695983_78192445 1p31.1 (0.49 Mb)	rs7515509	rs2133204	2.20 × 10^−1^	2.42 × 10^−2^	5.06 × 10^−2^	4.14 × 10^−3^	0.70	EUR
Moderate to severe hospitalized		rs7515509	rs2133204	2.43 × 10^−5^	3.23 × 10^−2^	1.02 × 10^−5^	1.16 × 10^−2^	0.96	EUR
Critically ill	Chr8:21773384_22270797 8p21.3 (0.49 Mb)	rs8192327	rs56390102	7.45 × 10^−5^	1.75 × 10^−5^	2.38 × 10^−1^	5.45 × 10^−2^	0.71	EUR
Moderate to severe hospitalized		rs8192330	rs56408342	2.49 × 10^−9^	3.51 × 10^−8^	9.94 × 10^−3^	1.38 × 10^−1^	0.85	EUR
Critically ill	Chr18:19748905_20248798 18q11.2 (0.49 Mb)	rs4800403	rs16967171	1.60 × 10^−5^	7.72 × 10^−5^	2.40 × 10^−3^	9.61 × 10^−3^	0.99	EUR
Moderate to severe hospitalized		rs4800403	rs3813126	2.05 × 10^−6^	7.75 × 10^−5^	1.64 × 10^−3^	6.02 × 10^−2^	0.94	EUR
SARS-CoV-2 reported cases		rs16967171	rs16967171	7.67 × 10^−6^	2.96 × 10^−3^	1.56 × 10^−5^	4.04 × 10^−3^	0.99	EUR
Critically ill	Chr8:21773384_22270797 8p21.3 (0.49 Mb)	rs56390102	rs56390102	8.60 × 10^−4^	1.11 × 10^−5^	2.55 × 10^−1^	1.83 × 10^−3^	0.74	SAS
Critically ill	Chr18:19748905_20248798 18q11.2 (0.49 b)	rs4800403	rs16967171	3.29 × 10^−4^	1.59 × 10^−3^	3.38 × 10^−3^	1.43 × 10^−2^	0.98	SAS
Moderate to severe		rs4800403	rs12958355	4.70 × 10^−11^	9.83 × 10^−19^	1.66 × 10^−3^	3.27 × 10^−2^	0.97	SAS
SARS-CoV-2 reported cases		rs16967171	rs12958355	5.44 × 10^−12^	2.04 × 10^−10^	2.47 × 10^−4^	7.27 × 10^−3^	0.99	SAS

**Table 3:** Bidirectional generalized summary data-based Mendelian randomization (GSMR) analysis showing the cause and exposure relationships at the three overlapping genetic loci.

Locus	Coordinates	$\hat{\beta }$ _zx_(forward-GSMR)__	$\hat{\beta }$ _ zy _y(reverse-GSMR)__	SE_zx(forward-GSMR)_	SE_zy(reverse-GSMR)_	*P*_Adj(forward-GSMR)_	*P*_Adj(reverse-GSMR)_
1p31.1	1_77695983_78192445	4.92	2.4 × 10^−3^	1.72	1.10 × 10^−2^	4.21 × 10^−3^	0.83NS
8p21.3	8_21773384_22270797	–0.61	0.10	0.28	2.71 × 10^−2^	3.19 ×10^−2^	2.20 × 10^−4^
18q11.2	18_19748905_20248798	2.07	0.16	0.44	3.02 × 10^−2^	2.30 × 10^−6^	1.72 × 10^−7^

Causal effect estimates for bidirectional GSMR—treating CAD and COVID-19 as both exposure and outcome across the three loci—are presented with their respective *p*-values and standard errors. Coordinates: Genomic location; $\hat{\beta }$_zx(forward-GSMR)_= Effect value for forward-GSMR where instrumental variables from CAD serving as exposures; $\hat{\beta }$_zy(reverse-GSMR)_: Effect value for reverse-GSMR where instrumental variables for COVID-19 were taken as exposure; SE_zx(forward-GSMR)_: Standard error of $\hat{\beta }$_zx(forward-GSMR)_; SE_zx(reverse-GSMR)_: Standard error of $\hat{\beta }$_zy(reverse-GSMR)_; ***P***_Adj(forward-GSMR)_: Benjamini–Hochberg (FDR) adjusted *p*-value for forward-GSMR; ***P***_Adj(reverse-GSMR):_ Benjamini–Hochberg (FDR) adjusted *p*-value for reverse-GSMR.

**Table 4:** Statistical associations of fine mapped lead signals or pleotropic signals with candidate genes prioritized in the gene level analyses through mBAT-combo at the three pleiotropic loci.

Gene	Genic Coordinates	Locus	Start SNP	End SNP	TopSNP	P_TopSNP_	Eig.	mBAT_Chisq_	P_mBAT-Combo_	P_mBAT_
PIGK	1_77088989_77219430	1p31.1	rs11162292	rs1963170	rs7515509	2.94 × 10^−12^	19	52.81	9.08 × 10^−6^	4.99 × 10^−5^
AK5	1_77282019_77559966	1p31.1	rs11162292	rs1963170	rs7515509	2.94 × 10^−12^	19	52.81	9.08 × 10^−6^	4.99 × 10^−5^
ZZZ3	1_77562416_77683419	1p31.1	rs11162292	rs1963170	rs7515509	2.94 × 10^−12^	19	52.81	9.08 × 10^−6^	4.99 × 10^−5^
USP33	1_77695987_77759852	1p31.1	rs11162292	rs1963170	rs7515509	2.94 × 10^−12^	19	52.81	9.08 × 10^−6^	4.99 × 10^−5^
DOK2	8_21908873_21913690	8p21.3	rs34802507	rs11777848	rs8192330	2.83 × 10^−7^	28	54.51	6.46 × 10^−6^	1.94 × 10^−3^
XPO7	8_21919662_22006585	8p21.3	rs34802507	rs11777848	rs8192330	2.83 × 10^−7^	28	54.51	6.46 × 10^−6^	1.94 × 10^−3^
NPM2	8_22024125_22036897	8p21.3	rs34802507	rs11777848	rs8192330	2.83 × 10^−7^	28	54.51	6.46 × 10^−6^	1.94 × 10^−3^
FGF17	8_22042398_22048809	8p21.3	rs34802507	rs11777848	rs8192330	2.83 × 10^−7^	28	54.51	6.46 × 10^−6^	1.94 × 10^−3^
DMTN	8_22048995_22082527	8p21.3	rs34802507	rs11777848	rs8192330	2.83 × 10^−7^	28	54.51	6.46 × 10^−6^	1.94 × 10^−3^
FHIP2B	8_22089150_22104911	8p21.3	rs34802507	rs11777848	rs8192330	2.83 × 10^−7^	28	54.51	6.46 × 10^−6^	1.94 × 10^−3^
NUDT18	8_22106874_22109419	8p21.3	rs34802507	rs11777848	rs8192330	2.83 × 10^−7^	28	54.51	6.46 × 10^−6^	1.94×10^−3^
HR	8_22114419_22133384	8p21.3	rs34802507	rs11777848	rs8192330	2.83 × 10^−7^	28	54.51	6.46 × 10^−6^	1.94 × 10^−3^
HRURF	8_22130604_22130708	8p21.3	rs34802507	rs11777848	rs8192330	2.83 × 10^−7^	28	54.51	6.46 × 10^−6^	1.94 × 10^−3^
REEP4	8_22138020_22141951	8p21.3	rs34802507	rs11777848	rs8192330	2.83 × 10^−7^	28	54.51	6.46 × 10^−6^	1.94 × 10^−3^
LGI3	8_22146830_22157084	8p21.3	rs34802507	rs11777848	rs8192330	2.83 × 10^−7^	28	54.51	6.46 × 10^−6^	1.94 × 10^−3^
SFTPC	8_22156913_22164479	8p21.3	rs34802507	rs11777848	rs8192330	2.83 × 10^−7^	28	54.51	6.46 × 10^−6^	1.94 × 10^−3^
BMP1	8_22165140_22212326	8p21.3	rs34802507	rs11777848	rs8192330	2.83 × 10^−7^	28	54.51	6.46 × 10^−6^	1.94 × 10^−3^
PHYHIP	8_22219703_22232101	8p21.3	rs34802507	rs11777848	rs8192330	2.83 × 10^−7^	28	54.51	6.46 × 10^−6^	1.94 × 10^−3^
POLR3D	8_22245133_22254601	8p21.3	rs34802507	rs11777848	rs8192330	2.83 × 10^−7^	28	54.51	6.46 × 10^−6^	1.94 × 10^−3^
PIWIL2	8_22275316_22357568	8p21.3	rs34802507	rs11777848	rs8192330	2.83 × 10^−^7	28	54.51	6.46 × 10^−6^	1.94 × 10^−3^
SLC39A14	8_22367278_22434129	8p21.3	rs34802507	rs11777848	rs8192330	2.83 × 10^−7^	28	54.51	6.46 × 10^−6^	1.94 × 10^−3^
PPP3CC	8_22440819_22541142	8p21.3	rs34802507	rs11777848	rs8192330	2.83 × 10^−7^	28	54.51	6.46 × 10^−6^	1.94 × 10^−3^
GATA6	18_22169589_22202528	18q11.2	rs9949157	rs12955964	rs4800403	1.27 × 10^−7^	16	55.79	1.43 × 10^−6^	2.63 × 10^−6^
CTAGE1	18_22413599_22417915	18q11.2	rs9949157	rs12955964	rs4800403	1.27 × 10^−7^	16	55.79	1.43 × 10^−6^	2.63 × 10^−6^

The results show the genomic range of each gene and locus (GRCh38 build), including the flanking SNPs, and the SNPs identified through association analysis. Gene: Likely casual genes; TopSNP: Lead SNPs; TopSNP Pvalue: *p*-value for TopSNP; Eig: No. of Eigenvalues; *χ*2: Chi-square (*χ*2) values; P_mBAT-Combo_: mBAT-combo *p*-value; P_mBAT_: mBAT *p*-value.

**Table 5:** Validation of prioritized candidate genes using bulk RNA-seq expression profiling data.

Gene	ID	baseMean	log2FoldChange	lfcSE	stat	*P*_DESeq2_	*P*_(Adj)-DESeq2_
DMTN	ENSG00000158856	2198.67	2.42	0.22	10.98	4.75 × 10^−28^	1.60 × 10^−25^
PIWIL2	ENSG00000197181	8.71	−2.18	0.89	−2.44	1.45 × 10^−2^	4.21 × 10^−2^

Gene: Candidate gene; ID: Ensembl ID for Gene; baseMean: Mean of normalized counts from all samples for Gene; log2FoldChange: Log2 Fold Change value of Gene; lfcSE: Standard error of Log2 Fold Change; stat: Wald statistic; ***P***_DESeq2_: DESeq2 ***p***-value; ***P***_(Adj)-DESeq2_: DESeq2 FDR adjusted *p*-value.

## Data Availability

Summary statistics generated by the COVID-19 HGI are publicly available on website (https://www.covid19hg.org/results/r7/, accessed on 28 April 2026), including per-ancestry summary statistics for African, admixed American, East Asian, European, and South Asian ancestries. CAD Summary statistics are available through the CARDIoGRAMplusC4D website (http://www.cardiogramplusc4d.org/, accessed on 28 April 2026) and the NHGRI-EBI GWAS catalog (https://www.ebi.ac.uk/gwas/, accessed on 28 April 2026), accession codes: GCST90132314. Expression profiling of PBMC data (GSE202805) derived from COVID-19 acute-severe patients (https://www.ncbi.nlm.nih.gov/geo/, accessed on 28 April 2026) and the 1000 Genomes Project (https://www.genome.gov/27528684/1000-genomes-project, accessed on 28 April 2026). The code for summary statistics, colocalization, and fine mapping (https://github.com/chr1swallace/coloc, accessed on 28 April 2026), HyPrColoc (https://github.com/cnfoley/hyprcoloc, accessed on 28 April 2026), and for PLINK (https://github.com/insilico/plink, accessed on 28 April 2026). Locuscomparer (https://github.com/boxiangliu/locuscomparer, accessed on 28 April 2026), locuszoomr (https://github.com/myles-lewis/locuszoomr, accessed on 28 April 2026), Mendelian randomization (GSMR2) (https://github.com/JianYang-Lab/gsmr2, accessed on 28 April 2026), DESeq2 (https://bioconductor.org/packages/release/bioc/html/DESeq2.html, accessed on 28 April 2026), and annotation tools (https://useast.ensembl.org/info/docs/tools/vep/index.html, accessed on 28 April 2026) are available on GitHub and in the R computing language (https://www.r-project.org/, https://github.com/rstudio/rstudio, accessed on 28 April 2026).

## Supplementary Materials

The following supplementary materials can be downloaded at: https://www.mdpi.com/article/10.3390/ijms27094132/s1.

## Abbreviations

The following abbreviations are used in this manuscript:

ABF	Approximate Bayes factors
ACS	Acute coronary syndrome
C2	Curated gene sets
C5	Ontology gene sets
CGP	Chemical and genetic
CPs	Canonical pathways
CAD	Coronary artery disease
CGPs	Chemical and genetic perturbations
EUR	European
fastBAT	Fast and flexible set-Based Association Test
FDR	False Discovery Rate
GSEA	Gene Set Enrichment Analysis
GO	Gene ontology
GWAS	Genome-Wide Association Study
GSMR	Mendelian randomization analysis
H	Hallmark gene set
HEIDI	Heterogeneity in dependent instruments
HGI	Host Genetic Consortium
HPC	High-performance computing
HPO	Human phenotype ontology
HyPrColoc	Hypothesis Prioritisation for multi-trait Colocalization
1KGP	1000 Genomes Project
LD	Linkage disequilibrium
LDL	Low-density lipoprotein
log2FC	Log2 fold change
mBAT	Multivariate set-based association test
MSigDB	Molecular signatures database
PP	Posterior probability
PBMC	Peripheral blood mononuclear cell
SNP	Single Nucleotide Polymorphism
SARS-CoV-2	Severe acute respiratory syndrome coronavirus 2
SAS	South Asian
VST	Variance stabilizing transformation
