# Supplementary material for: A Post-GWAS Analysis of the Shared Genetic Architecture Between COVID-19 and Coronary Artery Disease
Source: Int J Mol Sci. 2026 May 5;27(9):4132. doi: 10.3390/ijms27094132 (PMC13163323; doi:10.3390/ijms27094132)

## Slide 1
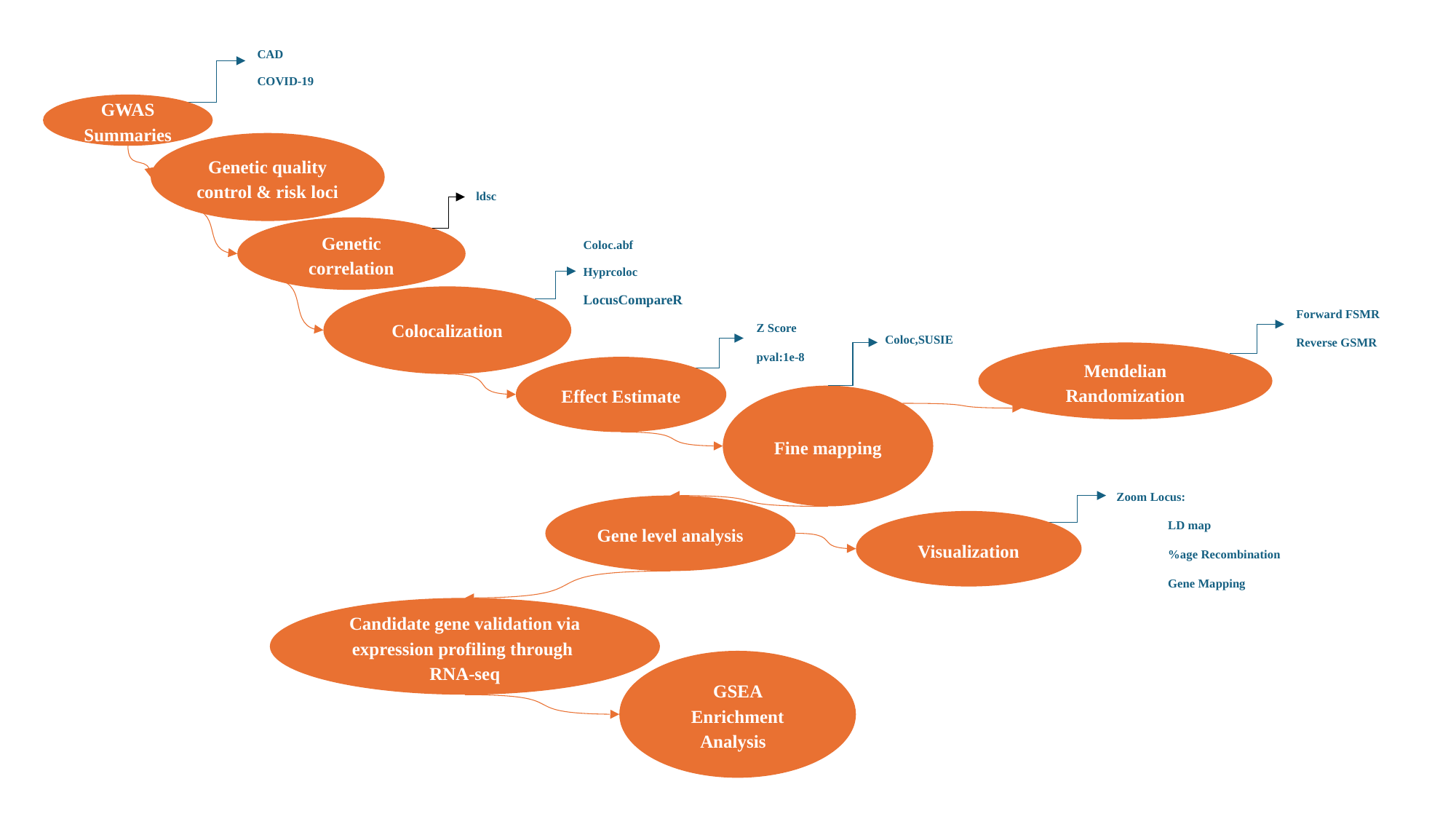

CAD
COVID-19
GWAS Summaries
Genetic correlation
Mendelian Randomization
Visualization
Candidate gene validation via expression profiling through RNA-seq
Effect Estimate
Fine mapping
Gene level analysis
Coloc.abf
Hyprcoloc
LocusCompareR
Z Score
pval:1e-8
 Zoom Locus:
LD map
%age Recombination
Gene Mapping
Genetic quality control & risk loci
Colocalization
GSEA Enrichment Analysis
ldsc
Forward FSMR
Reverse GSMR
 Coloc,SUSIE

## Slide 2
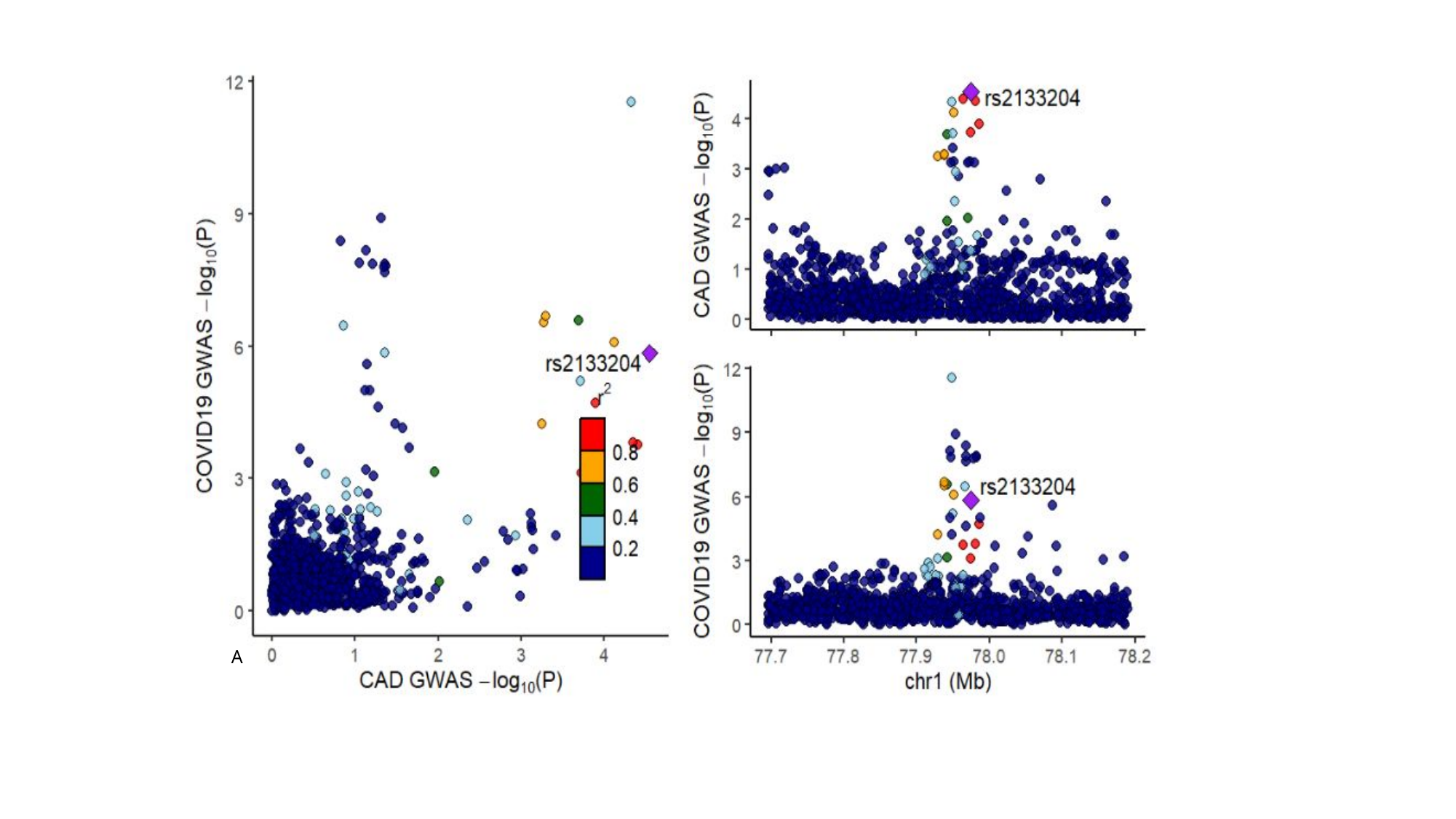

A

## Slide 3
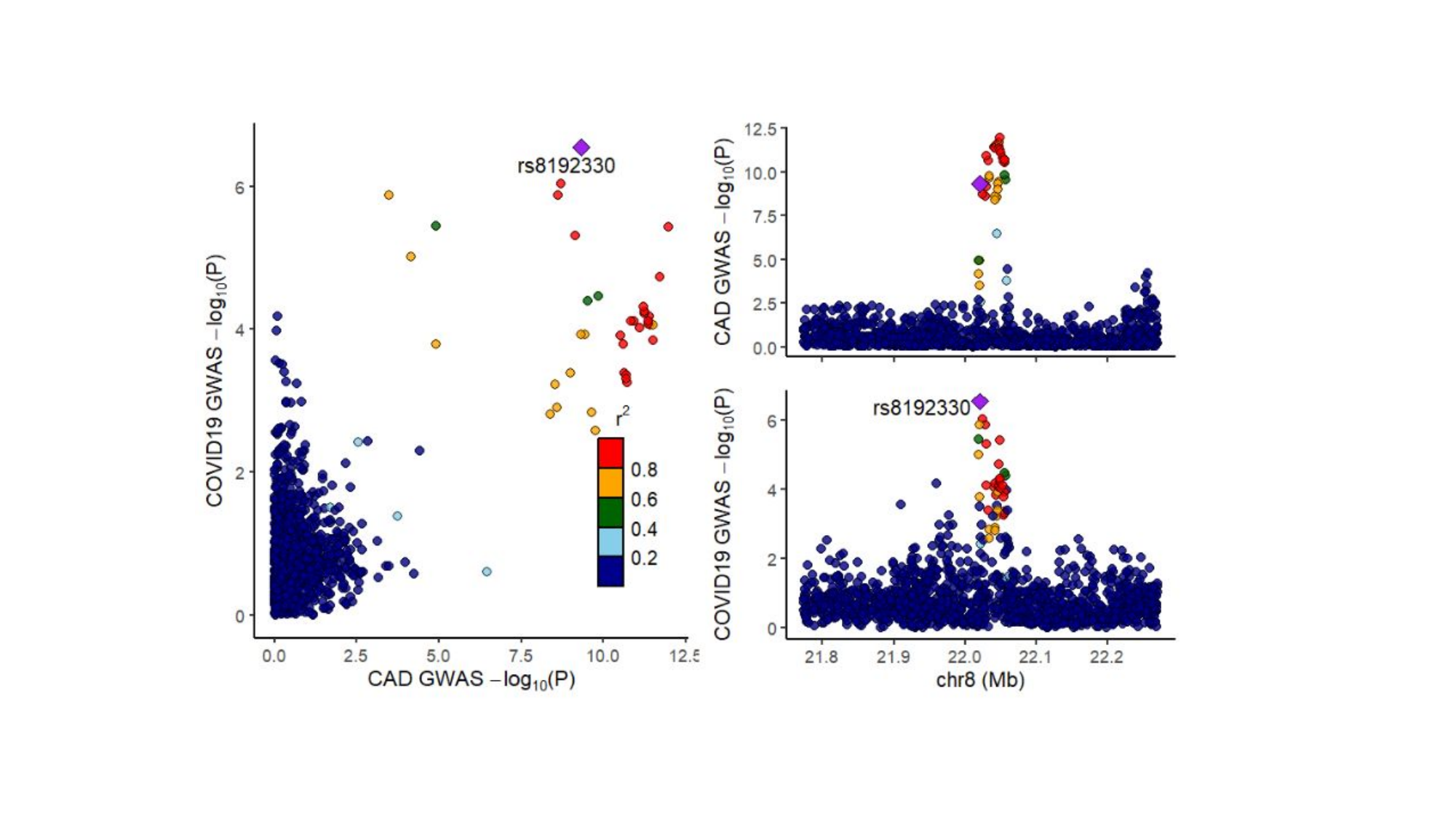

## Slide 4
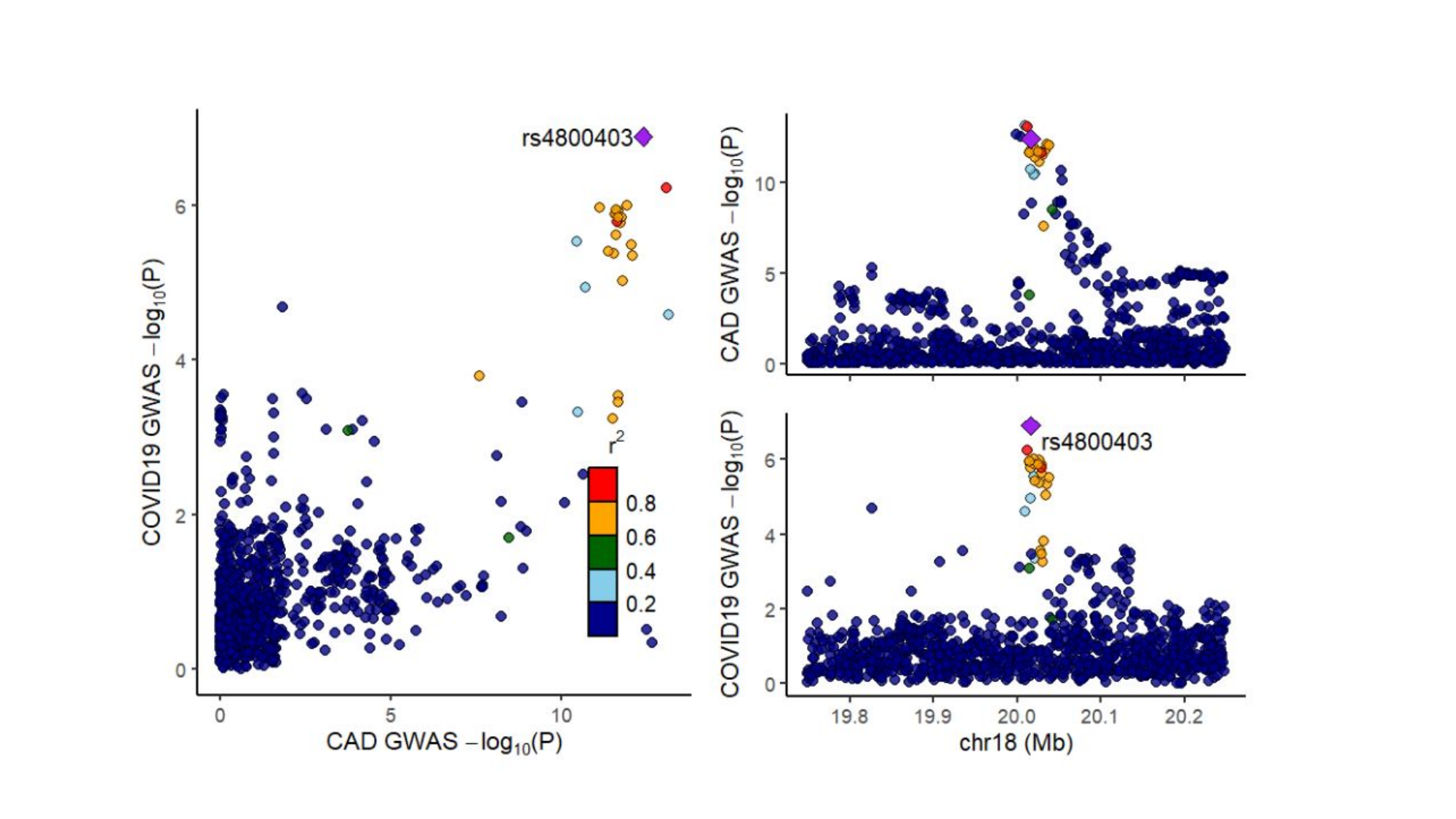

## Slide 5
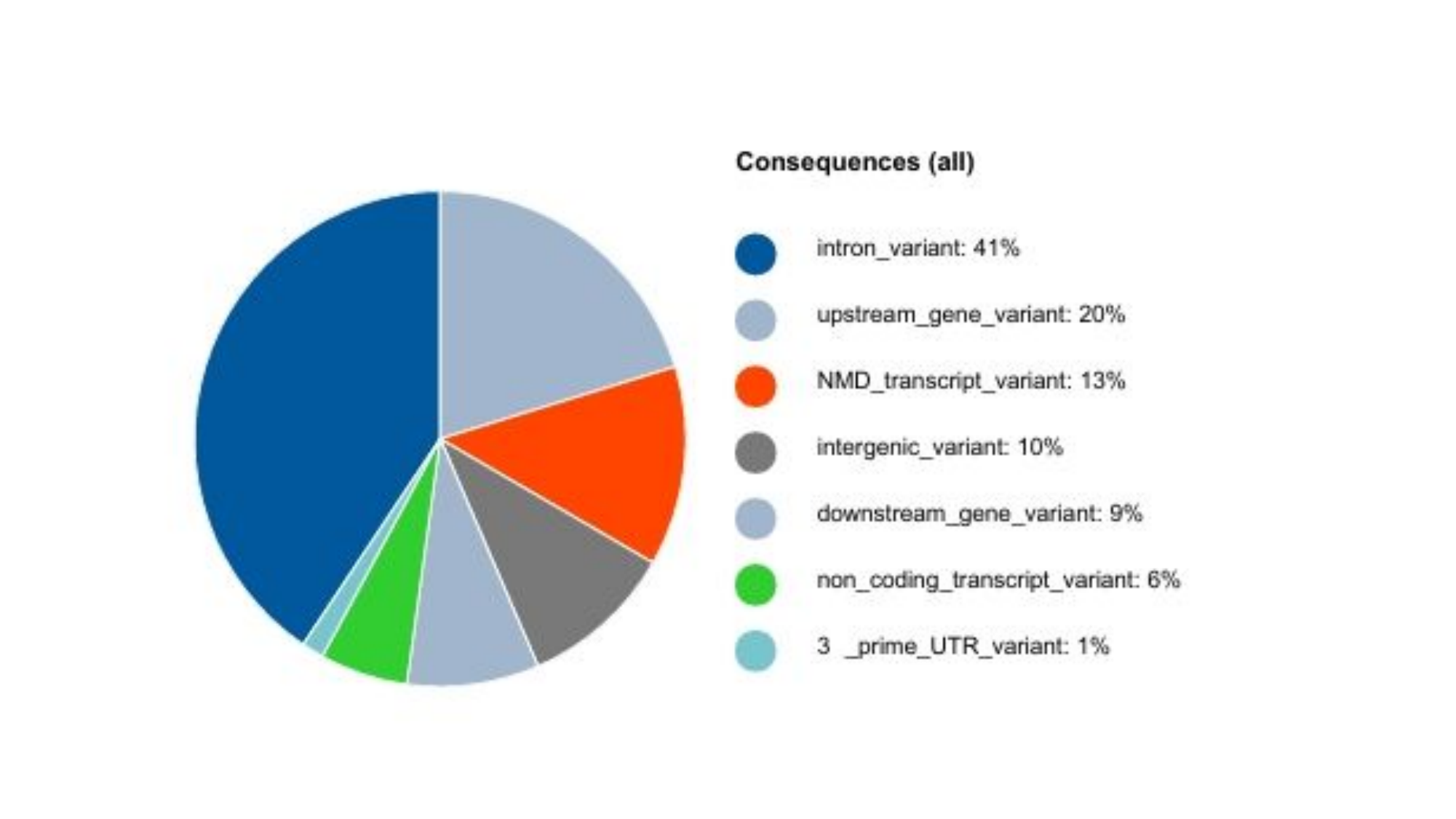

## Slide 6
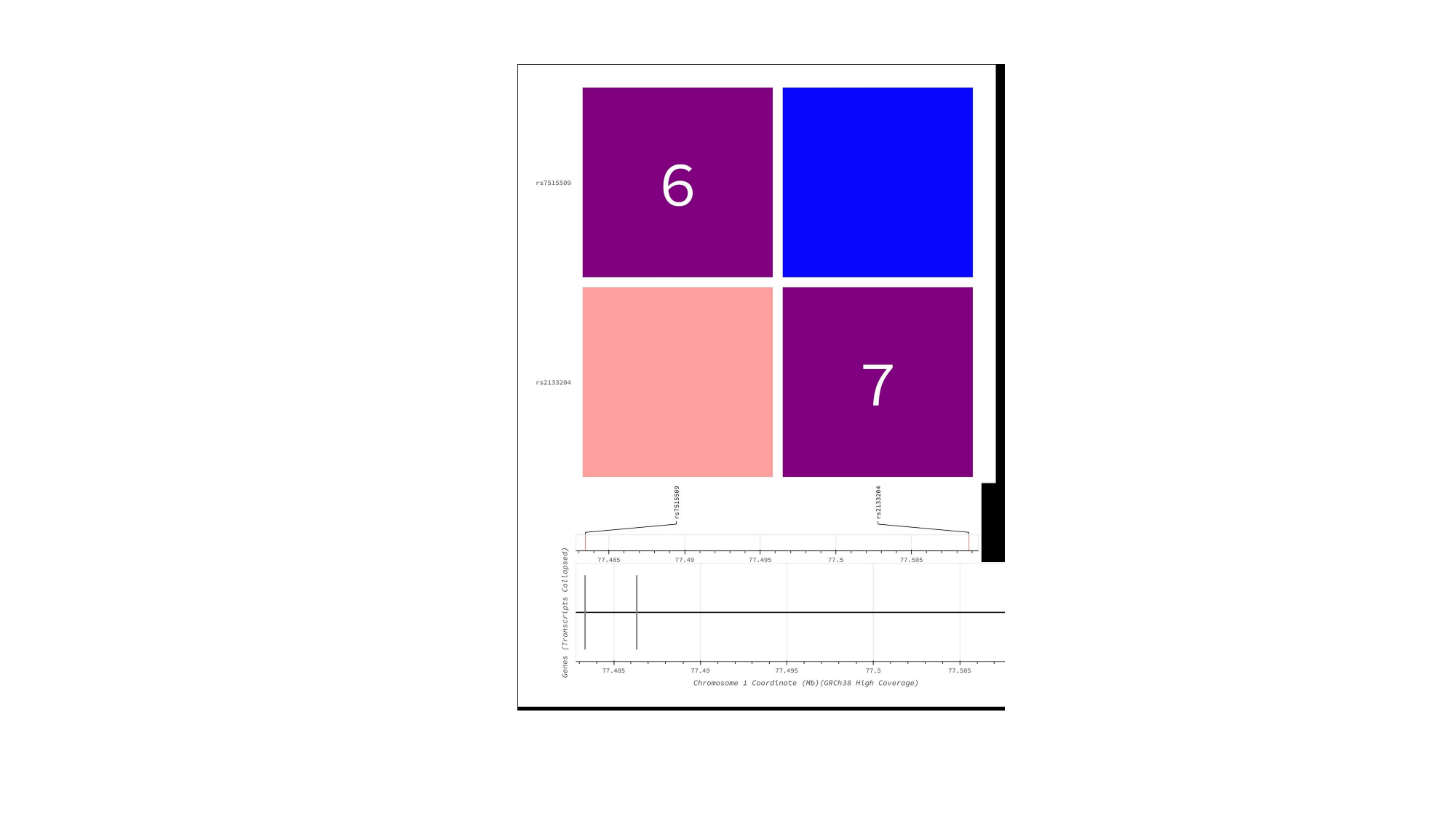

## Slide 7
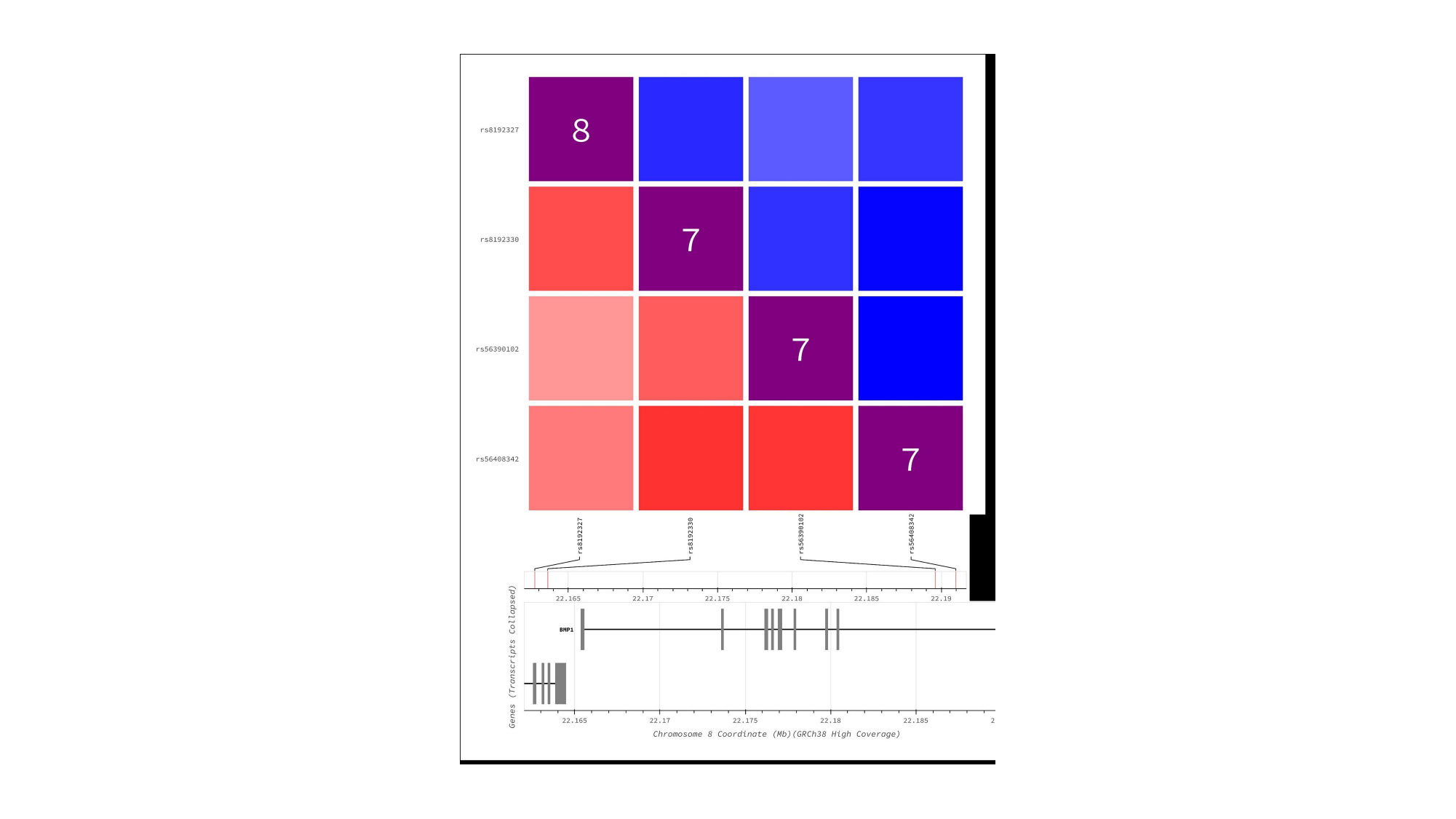

## Slide 8
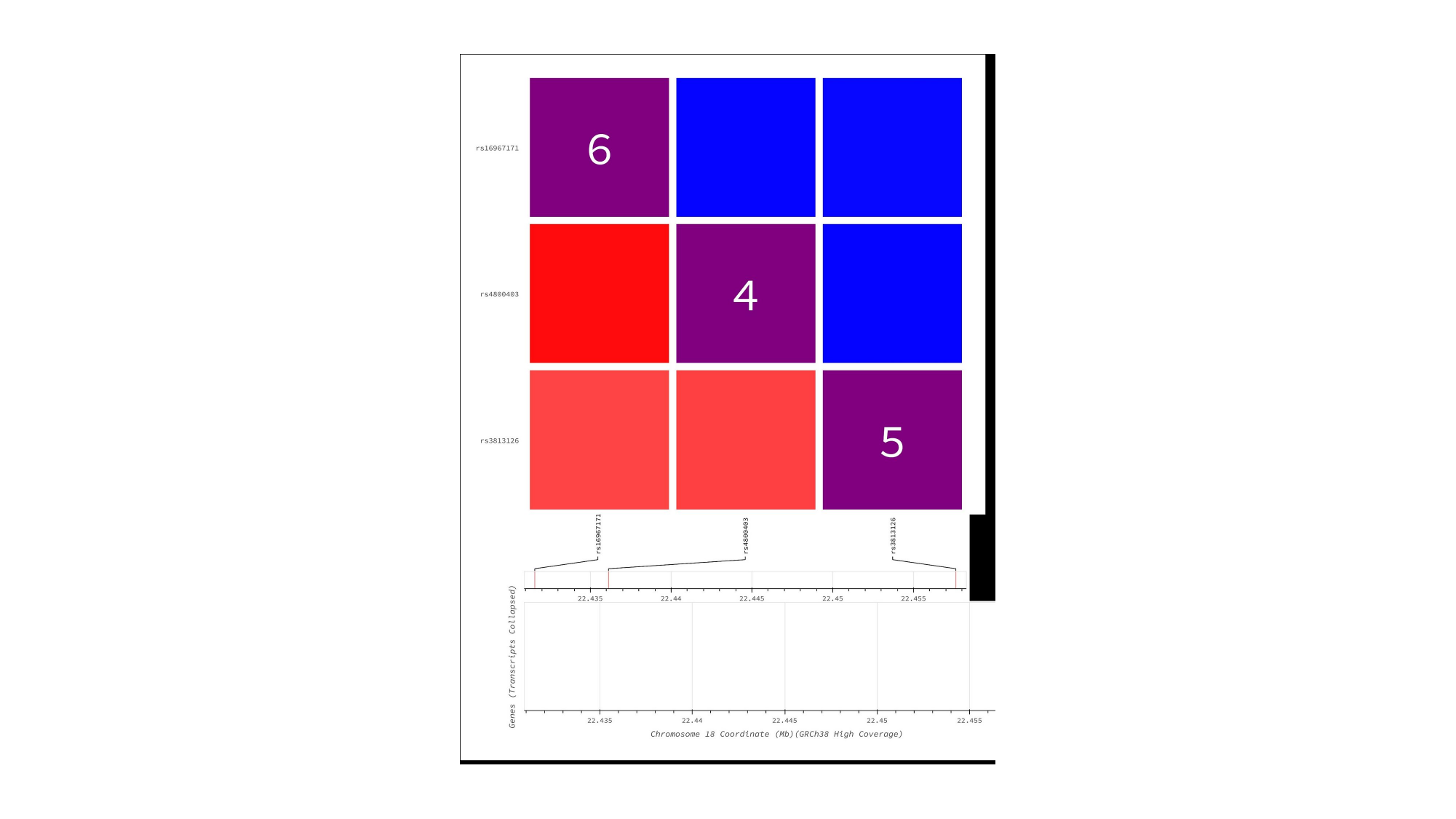

## Slide 9
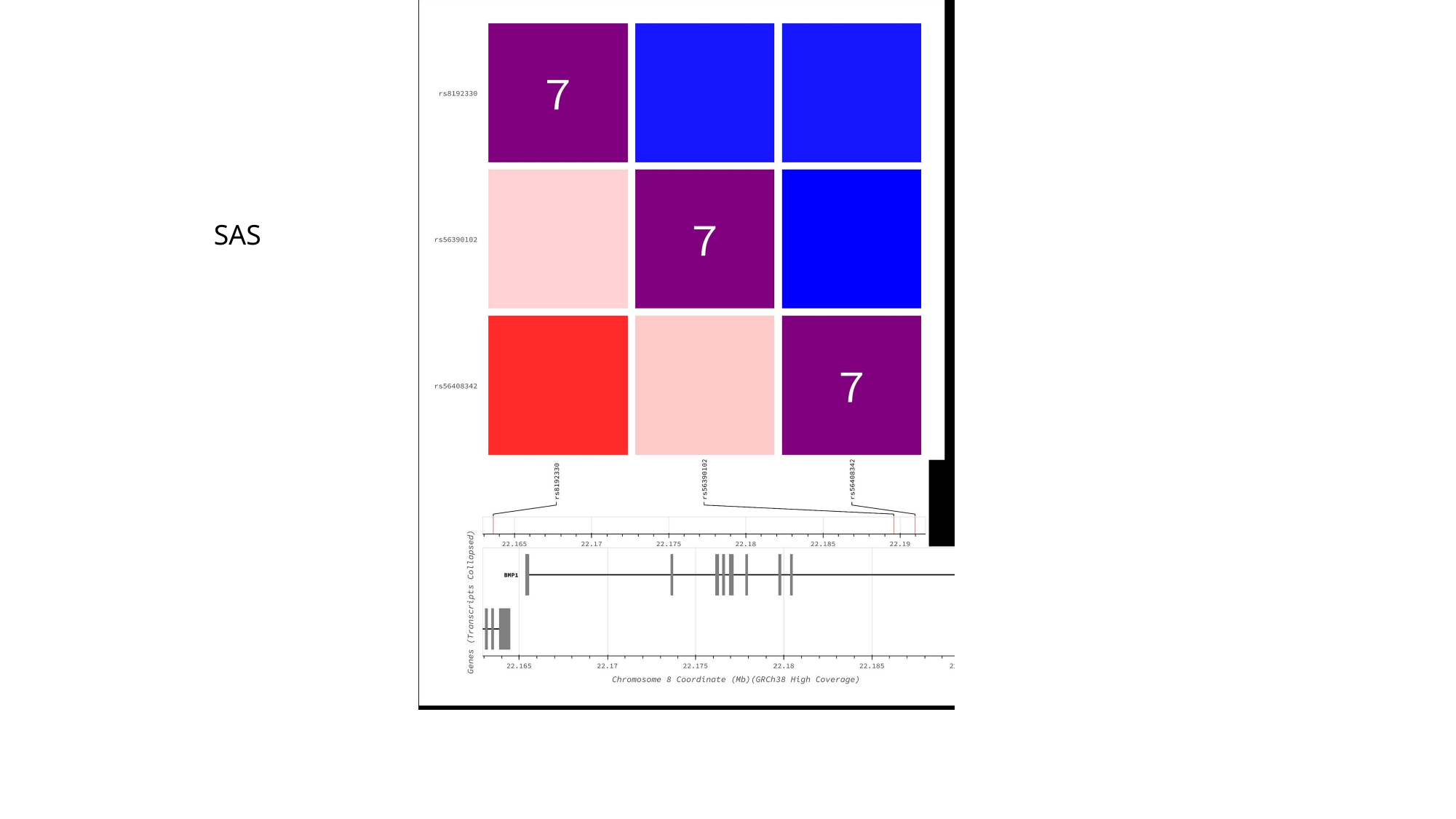

SAS

## Slide 10
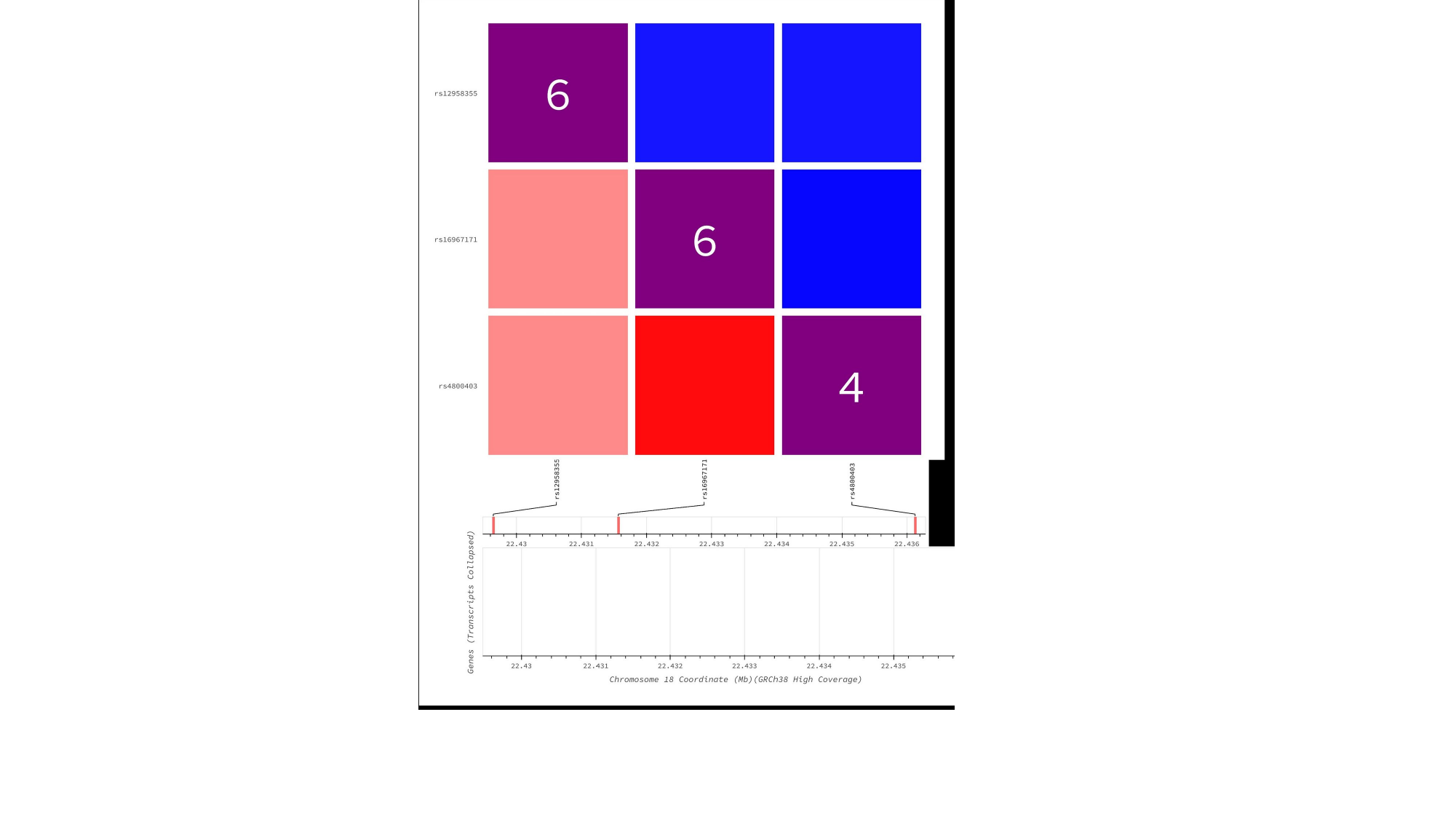

Supplement: Supplementary file 1 [file ijms-27-04132-s001.zip › Supplimentary Materials/Supplementary Figures.pptx]
